# Chemical Attachment of 5-Nitrosalicylaldimine Motif to Silatrane Resulting in an Organic–Inorganic Structure with High Medicinal Significance

**DOI:** 10.3390/pharmaceutics14122838

**Published:** 2022-12-18

**Authors:** Mirela-Fernanda Zaltariov, Mihaela Turtoi, Dragos Peptanariu, Ana-Maria Macsim, Lilia Clima, Corneliu Cojocaru, Nicoleta Vornicu, Bianca-Iulia Ciubotaru, Alexandra Bargan, Manuela Calin, Maria Cazacu

**Affiliations:** 1 Inorganic Polymers Department, “Petru Poni” Institute of Macromolecular Chemistry, Aleea Gr. Ghica Voda 41 A, 700487 Iasi, Romania; 2 Medical and Pharmaceutical Bionanotechnologies Laboratory, Institute of Cellular Biology and Pathology “Nicolae Simionescu” of the Romanian Academy, B.P. Hasdeu 8, 050568 Bucharest, Romania; 3 Centre of Advanced Research in Bionanoconjugates and Biopolymers, “Petru Poni” Institute of Macromolecular Chemistry, Aleea Gr. Ghica Voda 41 A, 700487 Iasi, Romania; 4 NMR Laboratory, “Petru Poni” Institute of Macromolecular Chemistry, Aleea Gr. Ghica Voda 41 A, 700487 Iasi, Romania; 5 Metropolitan Center of Research T.A.B.O.R, The Metropolitanate of Moldavia and Bukovina, 700066 Iasi, Romania

**Keywords:** 1-(3-aminopropyl)silatrane, 2-hydroxy-5-nitrobenzaldehyde, azomethine, protein binding, cytotoxicity

## Abstract

Two chemical motifs of interest for medicinal chemistry, silatrane as 1-(3-aminopropyl) silatrane (SIL M), and nitro group attached in position 5 to salicylaldehyde, are coupled in a new structure, 1-(3-{[(2-hydroxy-5-nitrophenyl)methylidene]amino}propyl)silatrane (SIL-BS), through an azomethine moiety, also known as a versatile pharmacophore. The high purity isolated compound was structurally characterized by an elemental, spectral, and single crystal X-ray diffraction analysis. Given the structural premises for being a biologically active compound, different specific techniques and protocols have been used to evaluate their in vitro hydrolytic stability in simulated physiological conditions, the cytotoxicity on two cancer cell lines (HepG2 and MCF7), and protein binding ability—with a major role in drug ADME (Absorption, Distribution, Metabolism and Excretion), in parallel with those of the SIL M. While the latter had a good biocompatibility, the nitro-silatrane derivative, SIL-BS, exhibited a higher cytotoxic activity on HepG2 and MCF7 cell lines, performance assigned, among others, to the known capacity of the nitro group to promote a specific cytotoxicity by a “activation by reduction” mechanism. Both compounds exhibited increased bio- and muco-adhesiveness, which can favor an optimized therapeutic effect by increased drug permeation and residence time in tumor location. Additional benefits of these compounds have been demonstrated by their antimicrobial activity on several fungi and bacteria species. Molecular docking computations on Human Serum Albumin (HSA) and M^PRO^ COVID-19 protease demonstrated their potential in the development of new drugs for combined therapy.

## 1. Introduction

The presence of silicon in a chemical structure induces some particularities as compared to carbon analogues. Thus, its use in drug design leads to some differences in terms of pharmacologic action and pharmacokinetic behavior [1]. First, silicon offers a large range of chemical reactivity, which is not available to standard carbon analogues. The increased lipophilicity allows the better permeability and biodistribution in vivo. The differences in electronegativity alter the bond polarization and increase the ability to interact with membrane cells. It was proved that intravenous administration of silicon-based drugs led to a higher clearance and a higher volume of distribution, compared with the carbon analogue compounds. Despite these benefits, there are only a few compounds, obtained by the substitution of a carbon atom with silicon in the core drug structures, which have entered the clinical trials but which are not marketed: *sila-budipine* with increased lipophilicity able to penetrate through the blood–brain barrier; *sila-haloperidol* with selectivity for dopamine receptors unlike the carbon analogue; *sila-fexofenadine* with a similar profile on histamine receptors’ binding; *sila-bexarotene* with a comparable response to the carbon analogue; *sila-venlafaxine* with an altered selectivity profile on noradrenaline and serotonin neurotransmitters unlike venlafaxine chloride; *sila-terfenadine*, an effective histamine antagonist, and two organosilane products, *flusilazole* (an antifungal agent) and *silafluofen* (an insecticide) [2,3]. A series of silicon-containing heterocycles obtained by silicon-for-carbon switch approaches [4] showed a similar lipophilicity with their carbon analogues, with a twofold half-life when administered in vivo due to plasma proteins’ binding ensuring a great distribution, but a higher instability against citochrome P 450 enzymes. Even so, it was highlighted that bioorganosilicon compounds are a complementary route toward a new class of lipophilic therapeutic drugs. Silicon-derived compounds, such as trimethylsilylpyrazoles, are inhibitors for p38 MAP kinase, activated especially in stress or by the immune response [5].

Silatranes, organosilicon compounds bearing a transannular dative bond between silicon and nitrogen atoms, and the pentacoordinate silicon atom, showed unique antibacterial, antifungal, antiviral, anti-inflamatory, and antitumor properties [6,7,8,9,10]. A marked antitumor activity was reported for 1-(alkylamino)silatranes [10]. The administration of 1-ethoxysilatrane has led to the resistance of the development of malignancies by stimulation collagen formation and connective stroma. Also, the antitumor activity of 1-(chloromethyl)silatrane evaluated in preclinical tests demonstrated the prevention of the development of neoplasms. The high antitumor activity was observed for 1-(β-cyanoethyl)silatrane, 1-vinylsilatrane, and N-γ-(silatrane-1-yl)propyl-N-allylthiocarbamide [10]. Other classes of silatrane derivatives were developed by the chemical transformation of attached amino groups, yielding imino (Schiff base) silatranes [11,12,13,14] or silatrane-1*H*-pyrrole-2-carboxamide [13], silatranes bearing the aryl(hetaryl)-substituted 1-azadienyl moiety [15], organosilatranes coupled with ferrocene through triazole [16], benzoyl substituted aminopropyl silatranes [17], or a ruthenium complex containing silatrane functional group [18], as promising building blocks for the design and synthesis of advanced materials and pharmaceuticals. These findings have evidenced that the silatrane scaffold-like structure offers multiple targets to exert its functionality and properties. Additional compounds obtained by its conjugation with precursors bearing specific functionality can lead to compounds able to retain at least both benefits.

On the other hand, nitro-containing drugs have attracted a special interest in the last years, the nitro group being activated in tumoral tissues by electron redistribution or endogenous generation [19,20,21]. Recently, it was established that some non-tumor drugs, such as nitroglycerin (a coronary vasodilator), nitroxoline (an antibacterial urinary agent), or nitro-fatty acids (anti-inflammatory agents), proved to be effective in cancer therapy [22,23].

Taking into account the high potential of nitro-functionality [24,25,26] and silatrane scaffold in medicinal chemistry, in the work reported here, 2-hydoxy-5-nitrobenzaldehyde was used as a precursor to synthesize a new silatrane imine derivative. The reported nitro silatrane derivative was structurally characterized by spectral and single crystal X-ray diffraction analysis and screened for its photophysical properties, hydrolytic stability, protein-binding ability, muco-adhesivity, antitumor, and antimicrobial activity. Furthermore, to gain an insight into its binding ability on proteins, molecular docking simulations on HSA and M^PRO^ (COVID-19 main virus protease) were carried out, highlighting the potential of these compounds in antitumor and anti-microbial/anti-viral therapies.

## 2. Materials and Methods

### 2.1. Materials

1-(3-aminopropyl)silatrane, SIL M, was prepared according to a procedure described in reference [11]. 2-hydroxy-5-nitrobenzaldehyde (Merck-Schuchardt) >98%, Acetonitrile (ACN) (Aldrich), chloroform (CHCl_3_) (Aldrich), dichloromethane (DCM) (Aldrich), dimethylformamide (DMF) (Aldrich), methanol (Aldrich), tetrahydrofuran (THF) (Aldrich) and dimethylsulfoxide (DMSO), Bovine Serum Albumin (BSA) (Sigma-Aldrich, Germany), Human Serum Albumin (HSA, 66.5 kDa, lyophilized powder, 96%) (Alpha-Aesar) were used as received.

### 2.2. Methods 

#### 2.2.1. Spectral Analysis

Carbon, hydrogen, and nitrogen content were determined on a Perkin–Elmer CHNS 2400 II elemental analyzer. IR spectra were registered on FT-IR spectrometer VERTEX 70 (Bruker) in transmittance mode, in the 400–4000 cm^−1^ spectral region, with a resolution of 4 cm^−1^ and 32 scans. ^1^H NMR and ^13^C NMR spectra were registered on a Bruker Advance NEO 400 MHz Spectrometer equipped with a 5 mm QNP direct detection probe and z-gradients. The spectra were recorded in DMSO-d6 and CDCL_3_-d1 at room temperature, and the chemical shifts are reported as δ values (ppm); *J* values were given in Hz using the solvent residual peak (2.51 for ^1^H and 39.5 for ^13^C in DMSO-d6 and 7.26 for ^1^H and 77.01 for ^13^C in CDCL_3_-d1) as reference. UV–Vis spectra were measured in acetontrile, chloroform, methanol, DMF, PBS pH 1.5, 2.6, 5, and 7.4, bovine serum albumin (BSA) solutions on a Specord 210 Plus spectrophotometer (Analytic Jena). Fluorescence spectra in solution were performed on a DuettaTM spectrometer (Horiba Ltd.) equipped with CCD detector and a light source (75 Mw Xenon) using 10 mm quartz cuvettes. Circular Dichroism spectra were measured on a Chirascan plus (Applied Photophysics) spectrometer, using a 10 mm light path cell. Data were recorded at 200–280 nm. All the spectral measurements were performed in PBS pH 7.4 at 22 °C.

#### 2.2.2. X-ray Crystallography

X-ray diffraction data were collected with an Oxford-Diffraction XCALIBUR E CCD diffractometer equipped with graphite-monochromated Mo*K*α radiation. The unit cell determination and data integration were carried out using the CrysAlis package of Oxford Diffraction [27]. The structure was solved by direct methods using Olex2 software [28] with the SHELXS structure solution program and refined by full-matrix least-squares on *F*² with SHELXL-2015 [29]. All H atoms bonded to carbon were entered in idealized positions (dCH = 0.97 Å) by using a riding model. CCDC 2175508 contains the supplementary crystallographic data for this contribution. These data can be acquired chargeless via www.ccdc.cam.ac.uk/conts/retrieving.html or from the Cambridge Crystallographic Data Centre, 12 Union Road, Cambridge CB2 1EZ, UK; (fax: (+44) 1223–336-033; or deposit@ccdc.ca.ac.uk).

#### 2.2.3. Cell Culture and Treatment

Human hepatocarcinoma (HepG2) and breast adenocarcinoma (MCF7) cell lines purchased from American Type Culture Collection (ATCC, Manassas, VA, USA), were grown in Dulbecco’s Modified Eagle Medium (DMEM) supplemented with 25 mM glucose (Gibco, ThermoFisher Scientific, Waltham, MA, USA), 10% fetal bovine serum (FBS)(Sigma-Aldrich, Darmstadt, Germany), and 1% Penicillin-Streptomycin from Gibco. For cell viability and cytotoxicity assessment, 7 × 10^4^/mL HepG2 or MCF7 cells were seeded in a 96- or 24-well culture plate (TPP^®^, Switzerland) for 48 h. Then, the cell medium was removed and changed by a fresh, colorless complete culture medium, supplemented with 0.3% DMSO (control cells) or SIL M and SIL-BS (concentrations in the range of 4.68 ÷ 300 μg/mL), and both cell lines were incubated for another 48 h. Then, the below protocols for cell viability/cytotoxicity assays were used.

#### 2.2.4. Cell Viability and Cytotoxicity Assays

##### XTT Assay

At the end of the incubation with compounds, the cells were washed three times with warm PBS and incubated for 2.5 h with 100 μL colorless complete medium supplemented with 0.25 mg/mL 2,3-Bis-(2-Methoxy-4-Nitro-5-Sulfophenyl)-2H-Tetrazolium-5-Carboxanilide (XTT) (Invitrogen, ThermoFisher Scientific) and 1.87 μg/mL N-methyl dibenzopyrazine methyl sulfate (PMS) (Acros Organics, ThermoFisher Scientific) [30]. The intensity of the orange color of the formazan derivative released by viable cells was measured at 450 nm against the complete medium supplemented with XTT-PMS in the absence of cells (blank), using the microplate reader spectrophotometer TECAN infinite M200Pro (Tecan Group Ltd., Switzerland). The cell viability was expressed as a percentage of control cells considered 100% viable.

##### Live/Dead Cell Assay

The cytotoxicity of SIL M and SIL-BS on HepG2 and MCF7 cells was assessed by the calcein-AM/propidium iodide (PI) staining method using the live/dead cell staining kit (SIGMA-Aldrich). The cell preparation and the staining protocol were performed as previously described [31]. The image acquisition was done using the 10 × objective of the Inverted Microscope Olympus IX81 equipped with fluorescence filters to detect green (calcein-AM) and red (PI) fluorescence indicative for viable and dead cells, respectively. The images were processed using the Image J software (National Institutes of Health (NIH), USA). Data were expressed as a percent of dead/ total (dead + viable) cell numbers.

#### 2.2.5. Bio- and Mucoadhesivity Tests

Bio- and mucoadhesive properties were investigated with a TA.XT Plus texture analyzer (Stable Micro Systems, Godalming, UK) on dialysis tubing cellulose membrane and porcine small intestine mucosa in PBS pH 7.4 at 37 °C. The force of detachment and the work of adhesion were calculated automatically by the software, after the calibration of standard parameters: the contact force of ≈ 1 mN, the contact time of 30 s, the speed of cylindrical holder of 1 mm/s. The registrations were performed in triplicate. 

#### 2.2.6. Antimicrobial Assay

Antimicrobial activity of the compounds was evaluated on three species of fungi: *Aspergillus niger* ATCC-16888, *Fusarium* ATTC-20327, *Penicillium chrysogenum* ATCC-11709, and two bacteria: *Bacillus* sp. ATCC-19986, *Pseudomonas aeruginosa* ATCC-27853, provided by American Type Culture Collection (ATCC), USA. The in vitro test was performed by the MIC test strip method according to the standard procedures (SR-EN 1275:2006 and NCCLS:1993). MIC strips are specific paper strips impregnated with predefined gradients of antimicrobial compounds concentration covering 15 double dilutions as for conventional MIC methods. The successive dilution procedure has been used to prepare the suspension of microorganisms. The final load of the prepared stock inoculum was 1 ×10^−4^µg/mL. Cultivation was performed by using 1:1 mixture of microorganism suspension and solution 1% DMSO of the compounds impregnated on paper strips and deposed on the solid medium in Petri dishes. After applying the paper strips on the seeded agar medium, the preformed gradient of the tested compounds was transferred to the agar and formed a stable, continuous, and exponential gradient of concentrations below the strip. A blank sample was also prepared in order to verify the influence of DMSO on the biological activity. The seeded plates were incubated for 24 h at 37 °C. The symmetrical ellipse of inhibition has been read directly from the scale at the point where the edge of the inhibition ellipse intersects the MIC test strip. MIC was calculated in μg/mL.

#### 2.2.7. Molecular Docking Computations

Computer-aided simulations related to the molecular docking were carried out by using the AutoDock VINA algorithm [32] encompassed in the YASARA program [32,33]. All calculations were done at the level of YASARA force field [33,34,35], and by using a Dell Precision Workstation T7910 for high-performance computations. The ligands employed for the molecular docking implied two organosilicon compounds: 1-(3-aminopropyl)silatrane (SIL M) and the Schiff base compound (SIL-BS). Initial structures of these ligands were taken from the crystallographic data (cif-files: CCDC 83251 for SIL M and CCDC2175508 for SIL-BS). The human serum albumin (HSA) and COVID-19 main protease (M^PRO^) were adopted as receptors for the molecular docking in this study. In this regard, the structures HSA and M^PRO^ receptors were downloaded from the Protein Data Bank (https://www.rcsb.org/(accessed on 20 October 2020)). The downloaded conformations (PDB ID: 1BJ5 and6LU7) were initially pre-processed in order to remove the crystallographic ligand and water molecules, as well as to add the missing hydrogen atoms. 

### 2.3. Synthesis of 1-(3-{[(2-hydroxy-5-nitrophenyl)methylidene]amino}propyl)silatrane, SIL- BS

A solution of 1-(3-aminopropyl) silatrane, SIL M, (0.100 g, 0.43 mmol) in chloroform (CHCl_3_) (5 mL) was added to a solution of 2-hydroxy-5-nitrobenzaldehyde (0.072 g, 0.43 mmol) in acetonitrile (ACN) (5 mL) in a round bottom flask. The color turned to yellow orange. The mixture was stirred at room temperature overnight, then filtered and left for crystallization at room temperature. The crystals appeared by slow evaporation as the solvents were filtered off, washed with cold acetonitrile/chloroform solvents mixture, and dried at room temperature. Crystals yield: 48 % (0.078 g). Calcd. for C_16_H_25_N_3_O_7_Si (M = 399.48 g/mol), %: C, 48.10; H, 6.31; N, 10.52. Found, %: C, 47.95; H, 6.11; N, 10.32. IR *ν*_max_(KBr), cm^−1^: 3466m, 3380m, 3356m, 3152w, 2924m, 2882m, 1658s, 1610vs, 1541s, 1483m, 1445m, 1406w, 1325vs, 1290s, 1274s, 1232s, 1188m, 1176m, 1163m, 1120s, 1090vs, 1051s, 1020s, 1006m, 938m, 910s, 833m, 798m, 779s, 756s, 715s, 685m, 627m, 578m, 547w, 526w, 505w, 482w, 403vw. ^1^H NMR (DMSO-*d_6_*, 400.13 MHz) δ (ppm): 14.07 (s, 1H, OH, **H16**), 8.71 (s, 1H, -CH=N-, **H10**), 8.42 (d, J = 3 Hz 1H, Ar-H, **H12**), 8.01 (dd, J = 3 Hz, 1 H, Ar-H, **H15**), 6.54 (d, J = 10 Hz, 1H, Ar-H, **H14**), 3.62 (t, J = 6 Hz, 6H, **H1**, **H3**, **H5**), 3.57(t, J = 7 Hz,2H, **H9**), 2.80 (t, J = 6 Hz, 6H, **H2**, **H4**, **H6**), 1.69 (s, J= 5 Hz, 2H, **H8**), 0.20 (q, J = 3 Hz, 2H, **H7**).^13^C NMR (DMSO-*d_6_*, 100.13 MHz) δ (ppm): 179.02 (**C16**), 166.89(-CH=N-, **C10**), 133.06 (**C12**), 132.94 (**C13**), 129.30 (**C15**), 123.21 (**C14**), 112.95 (**C11**), 56.59 (**C1**, **C3**, **C5**), 54.41 (**C9**), 49.88 (**C2, C4, C6**), 25.93 (**C8**), 13.87 (**C-7**). UV-vis (λ_max_(ε, M^−1^cm^−1^)): DMSO: 368 (27500), 410 (33796); acetonitrile: 358 (18450), 404 (20708); dichloromethane: 356 (28594), 408 (28784); methanol: 352 (16300), 392 (16426); THF: 354 (20436), 406 (21148).

## 3. Results and Discussion

**Scheme 1:**
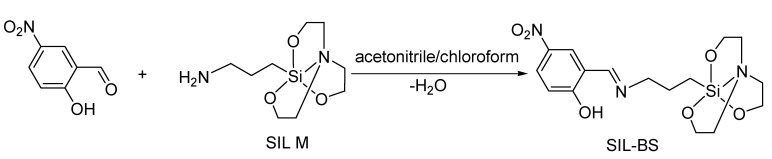
The synthetic pathway to prepare the silatrane derivative SIL-BS.

The synthesis of the silatrane derivative, SIL-BS, was performed by chemical modification of 1-(3-aminopropyl) silatrane, SIL M, by the condensation reaction with 5-nitrosalicylaldehyde in a 1:1 molar ratio (Scheme 1), in a mixture of acetonitrile/chloroform (ACN/CHCl_3_) solvents, followed by the stirring of the reaction mixture overnight and separation of the crystalline product as single crystals after the slow evaporation of the solvents at room temperature. The yellow–orange crystalline product was characterized by spectral methods in order to confirm its structure (FT-IR, NMR, UV-Vis), as well as by single crystal X-ray diffraction.

### 3.1. Structural Analysis

The structure of the SIL-BS derivative was firstly confirmed by IR spectroscopy, which showed the appearance of the characteristic bands for the azomethine group at 1610 cm^−1^ (corresponding to the enol tautomer) and at 1658 cm^−1^ (corresponding to the keto tautomer), at the same time with the disappearance of the characteristic bands of the amino groups in the structure of 1-(3-aminopropyl) silatrane at 3330 cm^−1^ and the carbonyl one from the aldehyde structure at 1664 cm^−1^. The other specific absorption bands of silatrane can be observed at 1188 and 1179 cm^−1^ (Si-C), 1163 and 1120 (υ_as_Si-O-), 1020 cm^−1^ (υ_s_Si-O-), 833 and 798 cm^−1^ (Si-C) (Figure S1).

The ^1^H NMR spectrum also demonstrated the structure of the SIL-BS compound by the presence of the chemical displacements specific to aliphatic protons between 0.20–3.62 ppm (**H1**–**H9**), aromatic ones from the aldehyde fragment between 6.54–8.42 ppm (**H12**, **H13**, **H15**, **H16**), and to the azomethine group at 8.71 ppm (**H10**), the ratio between them being in concordance with the proposed structure (Figure S2). The ^13^C NMR spectrum also showed the presence of the chemical displacements specific for the carbon atom related to the hydroxyl group at 173.02 ppm (**C16**), the carbon atom from the azomethine group at 166.89 ppm (**C10**) and from aromatic and aliphatic fragments at 133.06–112.95 ppm (**C11**–**C15**) and 56.58–13.87 ppm (**C1**–**C9**), respectively (Figure S3). The 2D correlation NMR spectra: H, H COSY, HSQC, and HMBC confirmed the assignments of the specific signals according to the structure (Figures S4–S6).

The values found by the elementary analysis fit well with those calculated based on the structure identified by the other techniques.

The presence of the ortho-hydroxyl group in the structure of the SIL-BS derivative may favor the formation of intramolecular hydrogen bonding interactions such as O-H…N and O…H-N, thus the appearance of keto-enol tautomerism [36].

In order to highlight this phenomenon, the UV-vis spectra were measured in solvents with different polarities: tetrahydrofuran (THF), dichloromethane (DCM), methanol (MeOH), acetonitrile (ACN), and dimethylsulfoxide (DMSO), the keto-enol tautomerism being also dependent on the H-bonding ability of the solvent, the presence, and the polarity of the substituents (in this case, the nitro group). Thus, the absorption spectra evidenced in all solvents the coexistence of keto-enol tautomers: enol form (S_1_← S_0_ (π*, π) transitions) at 367 nm (in DMSO) and keto tautomer (S_1_← S_0_ (π*, π) transitions) at 410 nm (in DMSO). The position of the absorption maxima found in DMSO was modified when solvents with lower polarity were used (Table 1). Thus, in DCM, the positions of the maxima were found at 356 nm (enol tautomer) and 408 nm (keto tautomer), respectively, while in methanol, a hypsochromic shift of the absorption maxima in dependence with the relative permittivity of the solvent was observed (Figure S7). However, one can observe that the interaction with solvents with H-bonding capability led to an enol-keto equilibrium toward the keto-tautomer, the values of molar absorption coefficients being higher for these species in solution (Table 1). All absorption spectra revealed that the keto-tautomer was stabilized by solvents similar with other Schiff base compounds [37].

### 3.2. X-ray Crystallography

**Figure 1:**
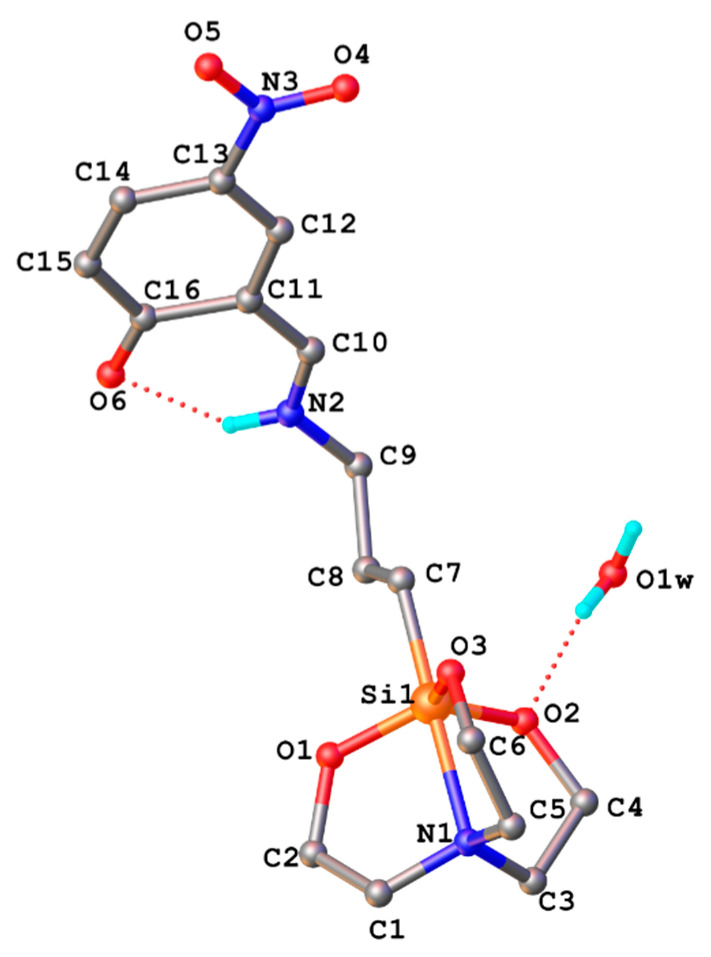
X-ray structure of the asymmetric part of SIL-BS.

**Figure 2:**
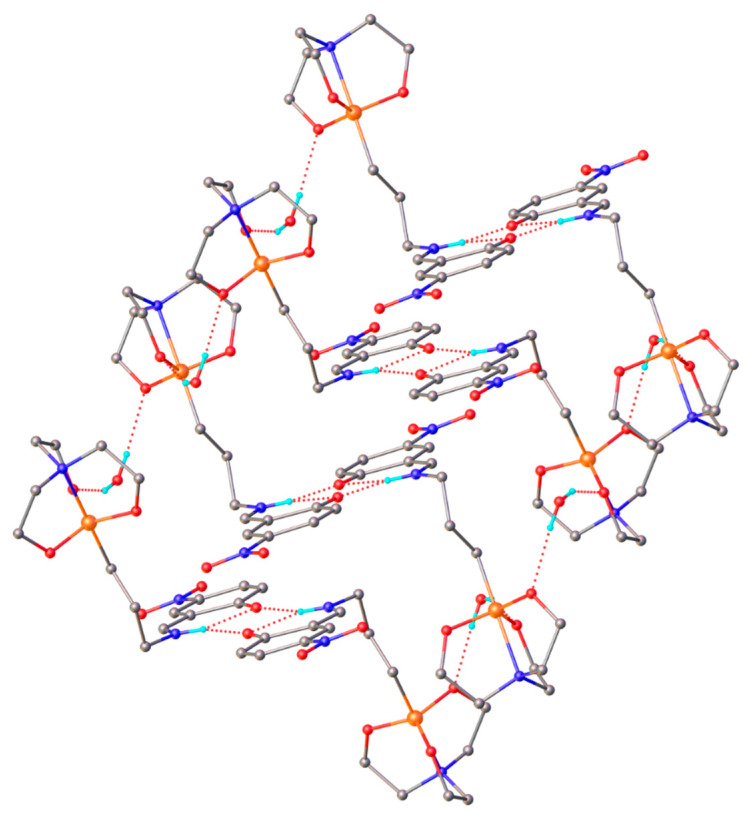
1D supramolecular chain in the crystal structure of SIL-BS formed by intermolecular hydrogen bonds N-H···O and O-H···O.

The X-ray study has revealed that the Schiff base SIL-BS has a molecular crystal structure consisting of the neutral entities [L] (Figure 1) and co-crystallized water molecule, in 1:1 ratio. The compound crystallizes in the non-centrosymmetric chiral space group (C2/c) in the monoclinic system. The molecule is in keto-amine form (in the solid state). The hydrogen atom H2 is located closer to the N2 than to the oxygen atom O6. The C16-O6 and C10-N2 bond distances are of high interest for this Schiff base to determine the enol-imine and keto-amine tautomerism. In Table 2, the main crystallographic data with refinement details for the studied compound are presented, while in Table S1 some selected bond lengths and angles are provided. The analysis of the structural data (Table S1) shows that O6-C16 (1.241(9) Å) is shorter than the distances found for C-OH in some similar compounds, 1.2981(3), 1.342(4), 1.351(5) Å [11,12]. Also, the value for N2-C10 (1.271(8) Å) is in agreement with the keto-amine form (Table 3) of the compound. The bond angles N2-C10-C11 (127.9(6) Å) and C10-N2-C9 (124.9(5) Å) are also consistent with the sp^2^ hybrid character for C10 and N2 atoms [38]. The structural characteristics of the compound SIL-BS show a remarkable similarity with those found for another Schiff bases containing a silatrane fragment in the structure [12]. The amine group acts as a donor to the oxygen atom of the ketone group. The molecular structure is stabilized by a strong intramolecular N2–H2A···O6 hydrogen bond and this leads to the formation of a six-membered ring with a N2-H2∙∙∙O6 bond length of 2.698(7) Å and an angle of 131.2°, thus locking the molecular conformation and eliminating conformational flexibility. Intermolecular interaction involves N–H···O and O*w*-H*w*···O interactions (Table 3, Figure 2), which stabilize the packing. The hydrogen bonding parameters are shown in Table 3. Water molecules act as bridging linkage for Schiff bases.

### 3.3. Hydrolytic Stability of SIL BS in PBS Media by Mimicking the Physiological Conditions

The stability of organosilicon derivatives in media mimicking the body conditions is an essential consideration for their applicability in medicine. Generally, silatranes are considered to be stable in air and in water or in more extreme acid environments, where hydrolysis can occur in dependence with the structure, the size, and the functionality [8,39]. The reactivity of Si-O-C, even if it is different in comparison with the corresponding carbon analogues (C-O-C bond), is thermodynamically very stable. However, the great resistance to hydrolysis can be very important in medical applications. More than that, the hydrolysis by-products of silatranes are mostly non-toxic and can be eliminated mainly by excretion [40].

The hydrolytic stability of 1-(3-aminopropyl)silatrane (SIL M) and its nitro-derivative SIL BS was studied by UV-vis and ^1^H RMN spectroscopy. SIL M is soluble in water and organic solvents, while the corresponding imine derivative is soluble only in organic solvents. The hydrolytic stability of SIL M is mainly attributed to the steric structure and the electron density distribution of the silatrane scaffold, the silicon atom being pentacoordinated, resulting in a very stable heterocycle. The stabilization of the heterocycle is supported by the ability of the lone electron pair of the nitrogen atom to interact with *d* orbitals of the silicon atom, resulting in a shorter bond distance Si-N [12]. The hydrolytic stability study of SIL-BS was performed in a PBS mixture with 1% DMSO at pH values of 1.5, 2.6, 5, and 7.4 (Figure S8). One can observe that the SIL-BS is stable in all pH media. At pH 7.4, there are two absorption maxima assigned to keto-enol tautomerisation equilibrium. By increasing the acidity of media from pH 5 to 1.5, one can notice a drastically diminishment of the band at 396 nm assigned to the keto tautomer favored in non-aqueous solvents, after 24 h, with the hypsochromic shifts by 47–55 nm of the absorption maximum at 367 nm (in DMSO). This is mainly due to the interaction with the medium and, as a consequence, the protonation on the nitrogen atom is supported even more by the strong electron-withdrawing nitro group in the structure [41]. At pH 5, the protonation at the nitrogen atom takes place more slowly; the spectral changes can be observed after 48 h, the phenolic tautomer being predominant in aqueos solution. At pH 7.4, the imine compound showed a slight hypsochromic shift of the maximum at 367 nm (enol tautomer by 7 nm—after 24 h and 17 nm after 7 days) and 410 nm (keto tautomer by 22 nm). Both maxima revealed a hypsochromic shift due to the interaction with the medium [42].

The stability of the imine compound was also highlighted by ^1^H NMR spectra registered in CDCl_3_ at room temperature and pH 1–4 after the addition of trifluoroacetic acid (TFA) (Figure S9). The main changes in acid media consisted of the chemical shifts of the azomethine and aromatic protons towards higher values by 0.6 ppm and of aliphatic protons by 0.2 ppm. The protonation also led to broad and overlapped signals especially on the aromatic domain, but the values of integrals remained unchanged. Thus, the azomethine proton (H10) shifted to 8.81 ppm; the aromatic protons: H12 to 8.65–8.57 ppm, H15 to 8.48–8.43 ppm, and H14 to 7.58–7.34 ppm. (Figure S9). In the aliphatic region, the signals assigned to H1, H3, and H5 are found at 4.19–3.64 ppm, those characteristic for H9 and H8 are observed at 2.01 and 0.14 ppm, respectively, while those attributed to H2, H4, H6 at 1.27–0.86 ppm. In time, in the ^13^C NMR spectrum of the Schiff base compound at pH 1, the disappearance of the chemical displacements assigned to the aliphatic atrane cycle cage can be observed. This is due to the slow hydrolysis of Si-O-C groups, by protonation at the oxygen atom and immediate nucleophilic attack at the silicon atom generating triethanolamine fragments in the solution (Figure S9) [43].

### 3.4. Evaluation of SIL-SB Effect on Cell Viability

**Figure 3:**
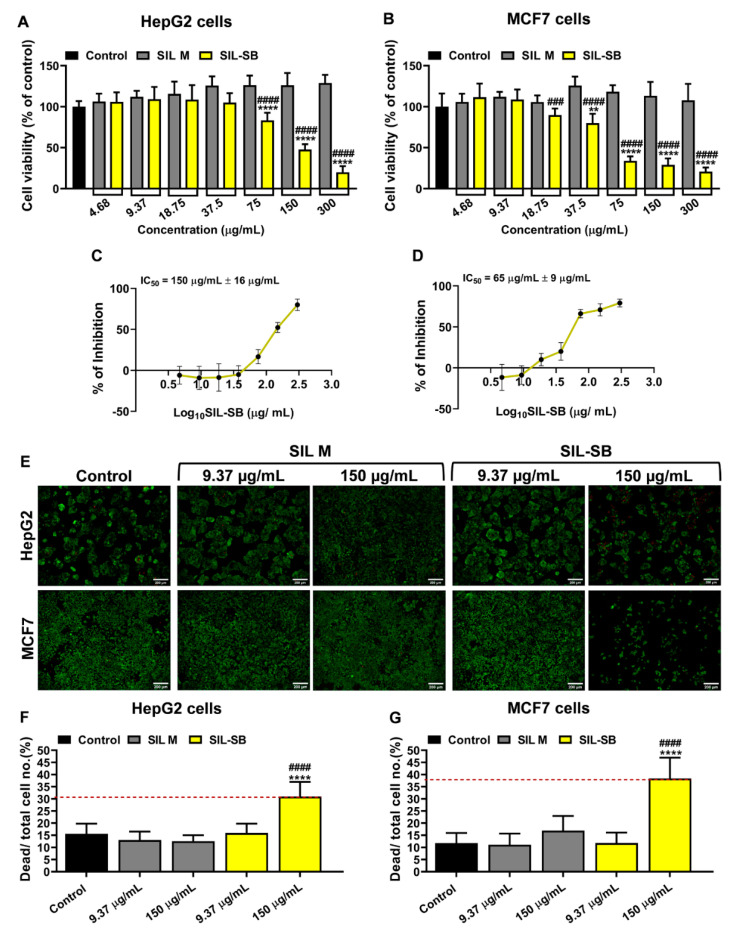
The viability of HepG2 (**A**) and MCF7 (**B**) cells, assessed by XTT assay. Dose response curves used to determine IC_50_ of SIL-BS for HepG2 (**C**) and MCF7 (**D**) cells. Cells were treated for 48 h with increasing concentrations (4.68 μg/mL to 300 μg/mL) of SIL M or SIL-BS. Merged Live/Dead cell images of HepG2 and MCF7 cells exposed to SIL M and SIL-BS for 48 h (**E**). Live cells were stained with calcein AM (green) and dead cells are detected with propidium iodide (red). Scale bar: 200 μm. The percentage of the dead to total cell number determined by Live/Dead cell assay for HepG2 (**F**) and MCF7 (**G**). Statistical significance: ** *p* < 0.01, **** *p* < 0.001 vs. control, ^###^ *p* < 0.001 and ^####^ *p* < 0.0001 vs. corresponding SIL M.

The effect of SIL-SB and its precursor SIL M on cell viability was analyzed after 48 h using two human carcinoma cell lines: hepatocarcinoma (HepG2 cells) and breast adenocarcinoma (MCF7 cells). The XTT assay showed that SIL M has good compatibility with HepG2 and MCF7 cells even at the highest concentration tested (300 μg/mL) (Figure 3A,B).

The HepG2 cells viability was reduced by about 20% when treated with 75 μg/mL Sil-BS, compared to control and SIL M-treated cells, and recorded a gradual decline after this concentration (≈ 50% at 150 μg/mL and more than 80 % at 300 μg/mL, *p* < 0.0001) (Figure 3A). On the other hand, the SIL-BS reduced the viability of MCF7 cells starting from 18.75 μg/mL compared to the corresponding SIL M concentration (*p* < 0.001) with a dramatic decrease observed at higher concentrations compared to both control and SIL M-treated cells (more than 70% for concentrations in the range 75–300 μg/mL, *p* < 0.0001) (Figure 3B). Also, the plotting of % of inhibition to log_10_ SIL-BS (µg/mL) revealed that SIL-BS induced a higher inhibitory effect on the viability of MCF7 tumor cells, half-maximal inhibitory concentration (IC_50_) being 65 µg/mL (Figure 3D), compared to IC_50_ of 150 µg/mL for HepG2 cells (Figure 3C).

The cytotoxicity induced by two concentrations of SIL-BS and SIL M, namely 9.37 and 150 µg/mL, on HepG2 and MCF7 cells was also analyzed by Live/Dead cell assay, which consists of staining of living cells in green (calcein-AM) and dead cells in red (propidium iodide) (Figure 3E). The Live/Dead cell staining data are in line with the XTT test results and support the higher sensitivity of MCF7 to the treatment with SIL-BS than HepG2 cells. The SIL M had no cytotoxic effects on HepG2 and MCF7 cells, yet, the SIL-BS at 150 µg/mL induced high toxicity on HepG2 and MCF7 cells, with a more pronounced effect on MCF7 cells (Figure 3F,G).

SIL-BS compound, the nitro silatrane Schiff base derivative, has specific antitumor activity on the MCF7 and HepG2 line and could be a good candidate for more detailed testing as a potential anticancer therapeutic agent. Also, the cytotoxicity on Normal Human Dermal Fibroblast (NHDF) cells revealed a low effect of SIL-BS at concentrations higher than IC_50_ on MCF7 and HepG2 and no effect for SIL M. Cisplatin, used for comparison, proved to be highly cytotoxic on both normal and cancer cell lines (Figure S10).

The search of new effective drugs with an unusual range of action in comparison with cisplatin, including the side effects associated with its administration, is a topical concern. Even at a higher concentration than the therapeutic ones of the cisplatin in specific tumor cells, such new drugs may exhibit different mechanisms of inhibition of tumor cells, consisting of different interactions with DNA than platinum drugs or involving some leading enzymes/proteins targets, thus reducing the drug resistance phenomenon [44]. Previously, it was found that 1-(alkylamino)silatrane can increase the antitumor activity of some drugs, such as cycliphosphamide, by direct influence on the fibroblast collagen and intercellular matrix organization [8].

**Scheme 2:**
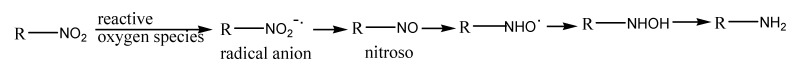
The bioreductive pathway of nitro group.

Based on these findings, the lack of antitumor activity of SIL M can be explained. Its application in combined therapies, such as wound dressing in local cancers, would also be good perspectives. On the other hand, the easy functionalization by imine bond formation of the silatrane scaffold can lead to the exposure to a targeted activity, even if these studies are insufficiently exploited and demonstrated. However, it has been proven that in medicinal chemistry, the nitro group is a unique functional group with a strong electron withdrawing ability and strong interaction with the biological media (proteins, enzymes, nucleic acids, amino acids, etc.) [45]. The nitro group can undergo enzymatic reduction promoting reactive species and inducing therapeutic effects. Recently, the nitro compounds have demonstrated anticancer activity by hypoxia induced effects, due to their bioreductive activation ability usually mediated by nitroreductases. The nitro reduction process involves the formation of a nitroso derivative, useful in the EPR effect on tumoral cells, as an intermediate toward primary amine compounds (Scheme 2) [46].

### 3.5. Protein Binding Ability

The most abundant protein in blood plasma is albumin, representing about 60% of the serum proteins. Albumin holds high biocompatibility and biodegradability, being water-soluble and stable at pH 4−9 and a temperature up to 60 °C, as well as nonimmunogenicity, being safely used in clinical administration. Its structure and conformation facilitate the interaction with many drugs, acting as a carrier, improving their bioadsorption, biodistribution, biotransformation, and acting as a shield from early elimination. At the tumor level, albumin can interact easily with the cells’ overexpressed receptors, ensuring the distribution of the drugs. Therefore, in situ binding by covalent or non-covalent interactions or encapsulation in albumin-based liposomes represent “targets” in cancer therapy. The mechanisms that allow the transport of albumin inside the cancer cells are receptor-mediated endothelial transcytosis and SPARC (secreted protein acidic rich in cysteine) [47]. These mechanisms lead to an enhanced permeation and retention effect (EPR) in tumors. Several studies revealed that the EPR effect can be improved by nitric oxide (NO), which is a vascular dilator, thus promoting an enhanced targeting and a higher impact in the suppression of tumor growth.

The ability of the compounds to bind proteins was investigated by spectroscopic methods (UV-vis, fluorescence, NMR and circular dichroism) and by molecular docking computations.

#### 3.5.1. UV-Vis and Fluorescence Spectroscopy

**Figure 4:**
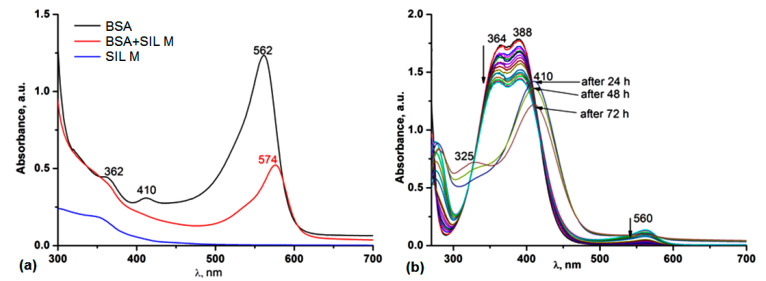
UV-vis spectra of SIL M (**a**) and SIL-BS (**b**) during and after titration with protein (BSA) solution 10%.

The spectroscopic techniques are frequently applied in the evaluation of serum protein binding ability in PBS media which mimics the body conditions (37 °C, pH 7.4). UV-Vis and fluorescence spectroscopy represents a facile way to monitor the interaction process of SIL M and SIL-BS with the BSA protein (of 10% concentration). The absorption spectra of SIL M and SIL-BS (1% DMSO) were recorded in PBS pH 7.4 (Figure 4a,b). SIL M has no absorption and emission maxima in the 300–750 nm spectral range. SIL-BS in PBS solution (1% DMSO) exhibits two absorption maxima, at 364 nm and 388 nm, and no emission maxima in the spectral range where protein emits. The protein solution has an absorption maximum at 560 nm, due to the aggregation of different amino acid fragments in the composition (especially tyrosine, tryptophan, and phenylalanine residues) (Figure 4a). By titration of the SIL M with protein solution, a bathochrome shift with 12 nm of the protein absorption maximum was observed, proving the silatrane binding to protein fragments. This shift occurs after 24 h. In the case of SIL-BS, by titration with a protein solution, a hypochromic shift occurs after each addition of 10μL BSA (10%). After 24 h, the absorption maximum of the protein disappears simultaneously with the bathochromic shift with 22 nm and hypsochromic shift with 40 nm of the absorption maximum of SIL-BS, which demonstrates the binding to the protein fragments (Figure 4b).

The fluorescence spectrum of the protein has a maximum emission at 338 nm, due to the tryptophan, tyrosine, and phenylalanine fragments present in its structure, tryptophan being the dominant intrinsic fluorophore of protein. The BSA protein exhibits two tryptophan residues: Trp 134 positioned at the surface and Trp 213 located inside the hydrophobic cavity of the protein. During the titration procedure, the concentration of the protein (10%) was maintained constant, while the concentration of SIL M and SIL-BS were varied from 10 μM to 200μM. By titration with SIL M solution 10^−3^ M, adding 20 μM SIL M, a slight decrease of the emission intensity was observed. Increasing SIL M concentration above this value, an increase of the emission intensity was observed, which was maintained even after 72 h from the addition of 100 μM SIL M. The maximum emission kept the initial position, indicating that no changes in the tryptophan residues’ environment occurred. 

**Figure 5:**
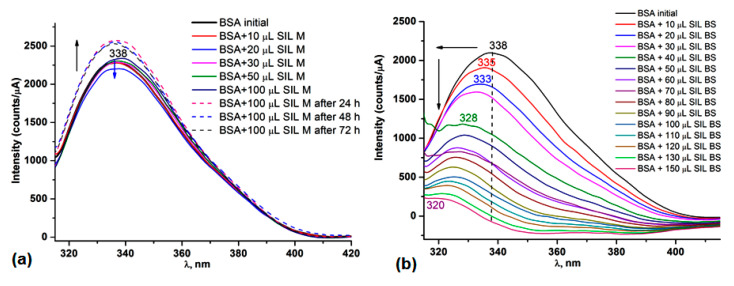
Emission spectra of the protein (BSA) during and after titration with SIL M (**a**) and SIL-BS (**b**) solutions 10^−3^ M.

By titration with the SIL-BS solution 10^−3^ M, the absorption maximum decreased simultaneously with the increase of the SIL-BS concentration. A hypsochromic shift with 18 nm of the emission maximum at a concentration of 200 μM of SIL-BS was observed (Figure 5). These hypochromic and hypsochromic shifts indicated that the conformation of protein was changed, suggesting that more hydrophobic residues of tyrosine are involved. The occurrence of the fluorescence quenching supports the binding process of SIL-BS [48].

**Figure 6:**
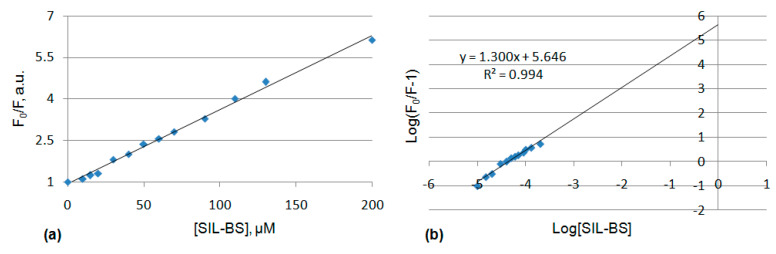
Stern–Volmer plot at λ_em_ = 338 nm obtained from steady-state measurements (λ_exc_=280 nm) in PBS pH 7.4 (1% DMSO SIL-BS) (**a**) and the double-log plots of SIL-BS quenching effect on BSA fluorescence (**b**).

The conjugation of BSA with different molecules is mainly described by two quenching mechanisms: static and dynamic, the static mechanism being characterized by the presence of a stable conjugated complex between the receptor (protein) and the ligand. In order to establish the quenching mechanism of binding SIL-BS to the BSA protein, the plotting data F_0_/F of BSA vs. [SIL-BS] was evaluated and the value of K_SV_ was estimated by the Stern–Volmer Equation (1):

F_0_/F= 1+ K_SV_[SIL-BS] (1)

where F_0_ is fluorescence intensity in the absence of SIL-BS, F is fluorescence intensity in the presence of SIL-BS at different concentrations [SIL-BS] of SIL-BS, and K_SV_ is the quenching (Stern–Volmer) constant. The linearity of the Stern–Volmer plots (Figure 6a) suggests a static quenching mechanism. The static quenching is also supported by the UV-vis spectra of the protein-SIL-BS (Figure 4b), where changes in the absorption maximum of protein were observed. In a dynamic (collisional) quenching, the excited states of the fluorophore are affected. 

The binding constant and the binding sites number can be described by the Equation (2):

log(F_0_/F − 1) = logK_b_ + *n*log[SIL-BS] (2)

where K_b_ is the binding constant, *n* is the number of the binding site of protein, and [SIL-BS] is the concentration of quencher. From this equation, the plotted data indicated a good linearity, the slope showed the *n* value, and the intercept with the Y-axis revealed the logK_b_. The value of *n* for BSA is 1.3, suggesting that there is one binding site of BSA for SIL-BS, while the binding constant (K_b_) is 4.43 × 10^5^ M^−1^ (logK_b_=5.646) (Figure 6b), proving a moderate binding of SIL-BS to protein [49].

#### 3.5.2. NMR Spectroscopy

In order to confirm the binding of the SIL-BS ligand to proteins, ^1^H NMR spectra were recorded after the addition of 3–4 drops of BSA (10%). After the addition of BSA, the ^1^H NMR spectrum of SIL-BS revealed some chemical shifts to higher values by 0.2 ppm of the aliphatic and aromatic protons, the presence of protein shielding the aliphatic proton signals (Figure S11). The binding to the protein by the azomethine and/or nitro groups is highlighted by the doubling of the signals assigned to aromatic protons (Figure S12). Over time, these changes are maintained. After 5 days of incubation with the protein at 37 °C, the ^1^H NMR spectrum also revealed the chemical shift specific for aldehyde proton at 10.1 ppm, while the ratio of the aromatic proton integrals also changed, supporting a partial hydrolysis to the azomethine groups accelerated by temperature.

#### 3.5.3. Circular Dichroism

The Human Serum Albumin (HSA) binding capacity of SIL M and SIL-BS was studied by the circular dichroism (CD) technique in the 200–280 nm spectral range. HSA has a remarkable structural similarity, sharing about 80% sequence homology and the degree of sequence and surface charge distribution with BSA, both of them being used in drug delivery.

HSA contains one tryptophan residue (Trp-214), while BSA has two tryptophan fluorophores (Trp-212 and Trp-134) [50]. The BSA protein includes 583 amino acids with a molecular weight of approximately 69.23 kDa, while HSA is composed of 585 amino acids and a molecular weight of 66.5 kDa. The secondary structure of BSA contains 67% helical structures, 10% turn, 23% extended chain configurations without any β sheets, consisting of three homologous domains (I, II, III), each possessing two sub-domains (A, B) [51], similar with HSA, which contains 66.7% α-helix, 8.2%-turn, and 20.6% random coils. Both proteins have numerous biochemical applications, being carriers for hormones, fatty acids, and other therapeutic agents and are involved in maintaining the physiological pH and osmotic pressure of the body [52].

CD spectroscopy is a useful tool in establishing the conformational changes and the α-helix content of proteins. CD spectra were recorded in PBS pH 7.4 using 0.7 mg/mL HSA protein solution and stock solutions of SIL M (73 mM) and SIL-BS (42 mM). The secondary structure of HSA is the main component which strongly absorbs circularly polarized light undergoing variations of n→π* and π→π* transitions. A pure α-helical structure of HSA exhibits two minimum absorptions at 208 and 222 nm assigned to π→π* and n→π* transitions, respectively, and a strong maximum at 190–193 nm. The β-sheet component exhibits a minimum at 216 nm specific for n→π* transitions and a maximum at 198 nm due to the π→π* transitions. The random coil structure exhibits minimum and maximum peaks opposite from those specific for α-helical and β-sheet segments [53]. Particularly, the quantification of helical content of a protein can be done by CD molar ellipticity at 222 nm, where the helical structure exhibits this minimum.

**Figure 7:**
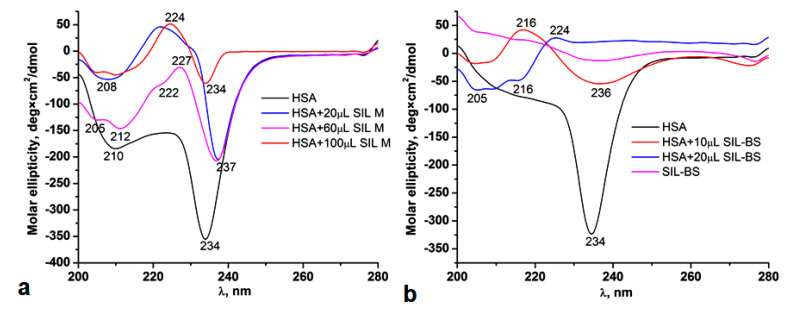
Circular dichroism spectra of SIL M (**a**) and SIL-BS (**b**) in PBS solution of HSA at pH 7.4.

In the registered CD spectra, the pure HSA protein exhibits two minimum peaks at 210 nm and 234 nm. By addition of increased concentrations of SIL M, the molar ellipticity decreases, yielding two broad minimums at 205 and 212 nm and one maximum at 224 nm assigned to protein helical structure and β-sheets. The minimum at 234 nm exhibited in all spectra indicates a perturbation and rearrangement of the secondary structure of the protein, suggesting the presence of random coils or an aggregation process as in case of surfactants. Other studies assigned this minimum to the interaction of tryptophan with other protein residues [54]. The random coil structure is also supported by the maximum at 224 nm. The minimum at 234 nm decreased by interacting with SIL M and disappeared by the addition of SIL-BS, which indicated that the binding to the protein induced some conformational changes, from disordered (random coil) to α-helix/β-sheets. This is supported by the appearance of the minimum peaks at 205 nm, 212 nm, and 222 nm, when the concentration of SIL M and SIL-BS increases (Figure 7).

#### 3.5.4. Molecular Docking Computations

**Figure 8:**
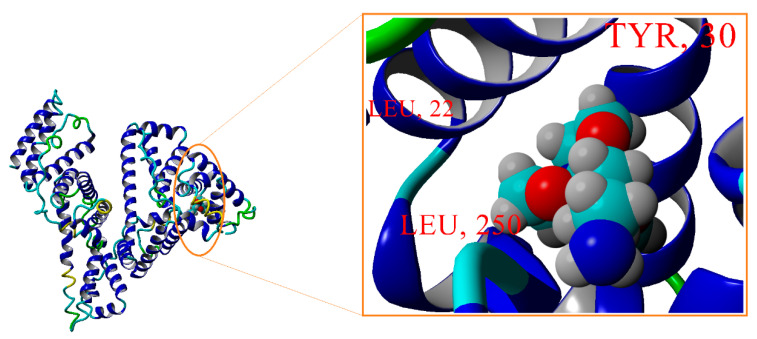
Molecular rendering of the best docked pose showing the interaction between HSA (receptor) and SIL M ligand; global docking view and zoom image.

**Figure 9:**
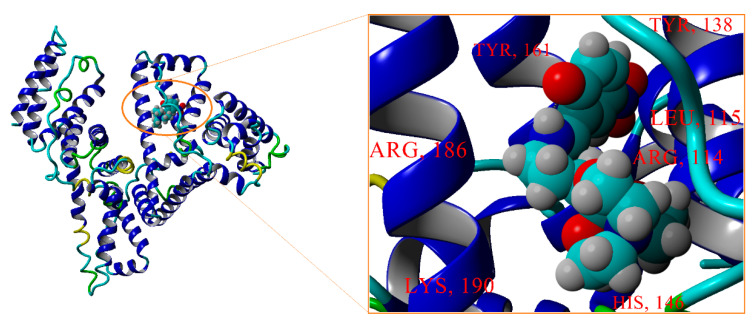
Molecular rendering of the best docked pose showing the interaction between HSA (receptor) and SIL-BS ligand; global docking view and zoom image.

During simulation, the number of docking trials was set to 100 in order to ensure reasonable search efficiency. The conformations of the ligands were treated as the rigid bodies, which were fixed at the level of the crystallographic structure (given in cif-file). This option of fixed atoms for ligands was adopted, since the YASARA program did not enable the automatic parameterization for the silicon atom. Hence, the empirical parameters for silicon atom were taken over from carbon (which is, from the isoelectronic standpoint, the nearest one). The binding modes of the ligand-receptor complexes were evaluated in terms of the relative energies of binding (*E_b_*, kcal/mol) and dissociating constant (*K_d_*, μM). Typically, the smaller values of these estimators indicate the stronger interaction between the receptor and ligand. Finally, the molecular docking outcomes were subjected to the clustering analysis by adopting a root-mean-square-deviation (RMSD) tolerance of 5.0 Å. The results of automated docking computations are shown in Figure 8 and Figure 9, for SIL M and SIL-BS ligand, respectively. As one can see, the binding pocket, in case of the ligand SIL M**,** is pinpointed in the sub-domains IA and IIA of HSA (Figure 8). In turn, for the ligand SIL-BS, the binding pocket is located in the sub-domain IB of HSA (Figure 9). Computational results revealed that the docked complexes HSA@SIL M (Figure 8) and HSA@SIL-BS (Figure 9) were stabilized by the hydrophobic interactions. 

Values of affinity estimators (relative energy *E_b_* and dissociation constant *K_d_*), as well as the contacting HSA-residues, are listed in Table 4. As reported, the number of contacting residues was equal to 14 and 12, for SIL-M and SIL-BS ligands, respectively. The dissociation constant is obviously diminished (*K_d_*=0.148 μM) for the docked system HSA@SIL-BS, when compared with the docked complex HSA@SIL M (*K_d_*=3.345 μM). According to these computational data, it turns out that the organosilicon compound SIL BS interacted much stronger with the receptor (HSA), if compared to the ligand SIL M.

### 3.6. The Bio- and Mucoadhesiveness

The mucoadhesive properties of SIL M and SIL-BS compounds had a great importance in the retention and residence time on a target location. Since tumor growth is ensured by a rich vascularization, the protein carrier’s function of the drugs will ensure a large distribution and availability to the local tumor; therefore, mucoadhesive drugs have emerged as specific platforms in cancer therapy. A mucoadhesive drug will accumulate and will exhibit a superior therapeutic effect in relation to efficacy/side effects, promoting a strong anti-tumor activity [55]. The bio-adhesion evaluation of the SIL M and SIL-BS indicated a strong interaction with synthetic (cellulose) membrane, a prototype material mimicking the cell membrane by the created pores in the surface. The force of adhesion with the synthetic membrane is approximately twice as large as with the small intestinal mucosa. The values of the adhesion force were 0.163 N in the case of SIL M and 0.118 N for SIL-BS. The work of adhesion was four times smaller in the case of SIL M (0.009 mJ), compared to SIL-BS, due to the higher solubility of SIL M which significantly reduces bio-adhesion (Table 5). Regarding the muco-adhesion, SIL M has a higher adhesion force (0.085 N) than SIL-BS (0.042 N), due to the higher solubility in PBS pH 7.4 and the high value of water absorption capacity of 50%, compared to 10% for SIL-BS. The water sorption is the first step involved in the muco-adhesion phenomenon, followed by the consolidation stage, in which the main processes are diffusion and interaction with the mucus constituents [56]. The work of adhesion values on porcine small intestine mucosa proved to be higher for SIL-BS. The contribution of intermolecular forces between the functional groups in the structure (OH, NO_2_ and -CH=N-) increases the adhesion with mucosa in the consolidation phase of the adhesion process.

### 3.7. Antimicrobial Activity

Since the discovery of the silatrane compounds by the Voronkov’research group, they proved a high and very specific biological activity due to their structural particularities. Thus, the spherical shape and high dipole moment of the molecule facilitate permeability through the cell membrane [57], while the versatility of the apical radical from silicon allows derivatization and tunability of biological activity [58]. It has been demonstrated that these compounds have a high reactivity, hydrolytic stability, and specific biomedical applications: pilotropic activity, pharmacologic role by antitumor, antibacterial, anti-inflamatory, anti-fungal activities, stimulating effects in animal production and seed germination [8], etc. Many other biological/pharmaceutical activities have been reported for various derivatives of silatranes: protein and collagen synthesis in cartilaginous tissues (1-ethoxysilatrane, methyl- and chloromethyl silatranes), reducing of the inflammation (chloromethyl and 1-ethoxysilatrane) and tumor growth (in renal cell carcinoma), stimulation of cell proliferation, reducing ulceration and accelerating the wound healing, anti-parasitic, anti-oxidation, anti-fungal, anti-bacterial, and anti-viral ones [7,8]. A remarkable biologic activity was first reported by Voronkov et al. in 1963 for 1-phenylsilatrane, which manifested a pronounced anti-fungal action on *Alternaria alternate* species at concentration lower than 10^−4^ M in water. The LD_50_ for this compound for white mice was about 0.35 mg/Kg, being considered extremely toxic. A concentration of 10^−3^% of 1-ethoxysilatrane was also found to inhibit some fungi species such as *Alternaria alternate, Penicillium ochrochloron, Botrytis cinerea*, and to stimulate some bacteria (*Bacillus subtilis*, *Bacillus mucilaginosus*) [8,59].

The antimicrobial activity of the 1-(3-aminopropyl)silatrane, SIL M, and the corresponding Schiff base derivative, SIL-BS, was investigated on three species of fungi: *Aspergillus fumigatus*, *Penicillium chrysogenum* and *Fusarium*, and two bacteria species: *Bacillus* sp. and *Pseudomonas* sp. (Table 6). The antimicrobial activity of SIL M was assessed in water, while for the SIL-BS derivative, DMSO solutions with different concentrations of 1% and 25% were used. SIL M showed a good antimicrobial activity, especially on fungi strains, while the 1% DMSO solution of SIL-BS showed a moderate antimicrobial action. At higher concentrations (25% DMSO), it does not show areas of inhibition on the microbial cultures.

The higher antimicrobial activity found for SIL M can be explained by the presence of the amino group, its higher basicity leading to an increased biocidal effect [60]. The cell membranes of microorganisms consist in an abundance of negative charges of the surface due to the presence of anionic liposaccharides and phosphatidylglycerol, thus explaining the higher interaction with SIL M which possesses the NH_2_ group. The simple condensation reaction of the amino group with an aldehyde, leading to an imine bond, reduced its activity. However, the moderate antimicrobial activity of SIL-BS can be explained by the increased lipophilicity by attaching the nitro-salicylaldehyde group [61]. Beside the influence of the structural functionalities (amino, nitro, imino, silatrane scaffold) on their biocidal activity, the lipophilicity of both samples must be considered, so that both hydrophobic and donor-acceptor interactions (hydrogen bonding) with the membrane lipid bilayers are involved in the antimicrobial mechanism [62].

The antimicrobial potential, along with the increased biocompatibility of SIL M and antitumor ones of SIL-BS revealed that they can be applied in the healing of wounds associated with microbial infections in cancer therapy, also taking into account the cellular proliferation of silatranes [7,8]. Silatrane derivatives possessing acyclovir groups also showed a specific anti-viral activity by inducing a characteristic immune response in HSV (Herpes Simples Virus). It was found that the silatrane fragment had a positive impact in the elimination of the virus from the organism by activation and proliferation of T lymphocite cells, its presence being beneficial as compared with the acyclovir alone [63].

The emergence of the pandemic transmissible SARS-CoV-2 infection produced by the COVID-19 virus led to the development of new anti-viral agents intended to be well tolerated by people with associated diseases: cancer, arterial hypertension, respiratory infections, diabetes, etc. Current treatment options for SARS-CoV-2 consist of traditional Chinese medicine combined with efficient drugs for SARS-CoV-1 and MERS-CoV (Chloroquine and Lopinavir/Ritonavir), which have been evaluated in clinical trials and some of them have received FDA approval for administration. Beside the specific antiviral activity, these drugs also have to possess first a minimal risk produced by the side effects, as well as a high binding affinity for target plasma proteins, ensuring a large bioavailability [64].

**Figure 10:**
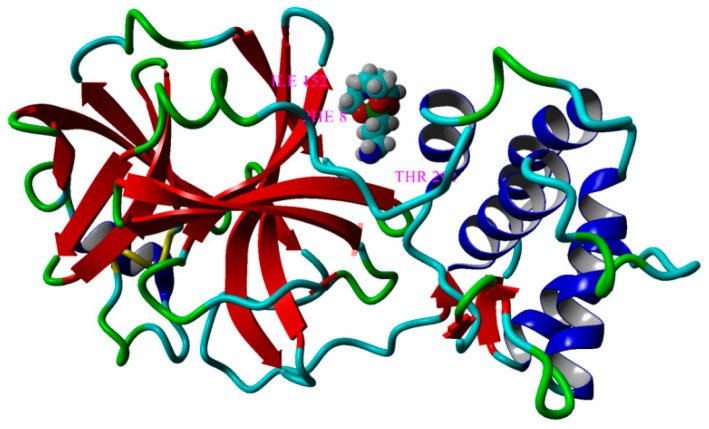
Molecular rendering of the best docked pose showing the interaction between M^PRO^ receptor (COVID-19 main protease) and silatrane SIL M.

Taking into account the above mentioned and given the water solubility, high biocompatibility and very good antimicrobial activity of 1-(3-aminopropyl)silatrane (SIL M), we have screened its antiviral capacity by molecular docking simulations on M^PRO^ (COVID-19 main virus protease) (PDB id: 6LU7 (https://www.rcsb.org/structure/6LU7 (accessed on 8 November 2020)). SARS-CoV-2 is an RNA virus exhibiting four structural proteins: spike, envelope, membrane, and nucleocapsid. The Main Protease (M^PRO^) also known as 3C-like protein is a 33.8 kDa cysteine protease having three domains. It has an essential role in the viral activity ensuring virus replication, so that its inhibition may be a target for antiviral drugs’ development [65]. A number of antiviral agents, such as Remdesivir, Cloroquine, and Hydroxychloroquine showed a considerable efficiency against the SARS-CoV-2 infection. The comparative docking studies of these agents highlighted that Remdesivir has the highest binding affinity toward M^PRO^ with a *E_b_* = −7.17 kcal/mol, followed by hydroxychloroquine and chloroquine with similar *E_b_* = −6.68 kcal/mol and −6.47 kcal/mol, respectively [66]. The silatrane compound, SIL M, also proved high inhibition ability on M^PRO^, estimated by docking studies, the *E_b_* was −5.794 kcal/mol close to the values of the above mentioned antiviral agents. Simulation results revealed that the docked complex M^PRO^@SIL M was stabilized by the hydrophobic interactions (Figure 10), and the silatrane (SIL M) contacted 14 residues from the receptor (Table 7). For this case, the affinity estimators were equal to *E_b_* = −5.794 kcal/mol and *K_d_* = 56.61 µM.

## 4. Conclusions

Chemical modification of 1-(3-aminopropyl)silatrane with 5-nitrosalicylaldehyde led to a new Schiff base derivative. The structure of the product was confirmed by elemental, spectral, and single crystal X-ray diffraction analyses. The nitro derivative demonstrated a high hydrolytic stability in PBS media of pH 7.4, and a moderate binding to proteins (BSA and HSA), as steady state fluorescence and circular dichroism methods attest. A 1:1 interaction with BSA involving the tryptophan residues of protein and a binding constant of 4.43 × 10^5^ M^−1^ was found. The estimated dissociation constant of nitro silatrane derivative (K_d_= 1.48 × 10^5^ M^−1^) for HSA by molecular docking simulations demonstrated a stronger interaction than 1-(3-aminopropyl)silatrane (K_d_= 3.345 × 10^5^ M^−1^) and different binding pockets. The nitro silatrane derivative proved to be more muco-adhesive and possessed a higher cytotoxicity than 1-(3-aminopropyl)silatrane. Instead, the latter has a higher antimicrobial activity and ability to bind on an M^PRO^ receptor (COVID-19 main protease) with a binding affinity comparable to that of chloroquine and hydroxychloroquine antiviral drugs. Moreover, the nitro silatrane derivative induced higher cytotoxicity on human breast adenocarcinoma (MCF7 cells) with IC_50_ of 65 µg/mL than on hepatocarcinoma (HepG2 cells). The higher selectivity on MCF7 cell lines of nitro silatrane derivative was also confirmed by the Live/Dead cell staining assay. Thus, our results suggest that the nitro silatrane Schiff base derivative could be a good candidate for more detailed testing as a potential anticancer therapeutic agent in order to elucidate if the cytotoxic mechanism occurs by an in situ nitroreduction pathway.

## Figures and Tables

**Table 1:** Spectral characteristics of SIL-BS in solvents with different polarities.

Solvent	Solvent Characteristics		SIL-BS	
	Relative Permittivity	Polarity Index (Pi)	λ_max_Enol (ε)	λ_max_Keto (ε)
DMSO	47.24	7.2	368 (27,500)	410 (33,796)
ACN	36.64	5.8	358 (18,450)	404 (20,708)
MeOH	33.00	5.1	352 (16,300)	392 (16,426)
DCM	8.93	3.1	356 (28,594)	408 (28,784)
THF	7.52	4	354 (20,436)	406 (21,148)

λ_max_—maximum of absorption; ε—molar extinction coefficient.

**Table 2:** Crystal data and details of data collection for SIL-BS.

Compound	SIL-BS
empirical formula	C_16_H_25_N_3_O_7_Si
Fw	399.48
space group	C2/c
a [Å]	23.484(2)
b [Å]	18.2616(18)
c [Å]	9.4033(10)
α [°]	90
β[°]	99.431(9)
γ [°]	90
V [Å^3^]	3978.2(7)
Z	8
ρ_calcd_[g cm^−3^]	1.334
Crystal size [mm]	0.45 × 0.15 × 0.1
T [K]	293(2)
μ [mm^−1^]	0.160
2Θ range	3.516 to 50.048
Reflections collected	8645
Independent reflections	3513 [Rint = 0.0516]
Data/restraints/parameters	3513/0/247
R_1_^[a]^	0.0944
wR_2_^[b]^	0.2847
GOF ^[c]^	1.042
Largest diff. peak/hole / e Å^−3^	0.66/−0.36

^[a]^*R*_1_ = ∑║*F*_o_│ − │*F*_c_║/∑│*F*_o_│, ^[b]^ *wR*_2_ = {∑[*w* (*F*_o_^2^ − *F*_c_^2^)^2^]/∑[*w*(*F*_o_^2^)^2^]}^1/2^, ^[c]^ GOF = {∑[*w*(*F*_o_^2^ − *F*_c_^2^)^2^]/(*n* − *p*)}^1/2^, where *n* is the number of reflections and *p* is the total number of parameters refined.

**Table 3:** Hydrogen Bonds for SIL-BS.

D-H-A	d(D-H)/Å	d(H-A)/Å	d(D-A)/Å	D-H-A/°
N2-H2-O6 ^1^	0.86	2.13	2.839(7)	139.9
N2-H2-O6	0.86	2.05	2.698(7)	131.2
O1*w*-H1*w*A-O2	0.85	2.03	2.866(6)	167
O1*w*-H1*w*B-O3^2^	0.85	2.1	2.910(6)	157.7

^1^ 1 − *x*, 1 − *y*, 1 − *z*;^2^+ *x*, 1 − *y*, ½ + *z.*

**Table 4:** Contacting receptor residues in docked complexes (by studied ligands SIL-M and SIL-BS), and calculated affinity estimators (relative binding energy and dissociation constant).

Docked complex (Receptor@Ligand)	No. of Contacting Residues	Contacting Residues in the Receptor (HSA)	*E_b_* (kcal/mol)	*K_d_* (µM)
HSA@SIL M	14	LEU22, VAL23, ALA26, PHE27, TYR30, VAL46, LEU66, HIS67, PHE70, ASN99, ASP249, LEU250, LEU251, GLU252	−7.470	3.345
HSA@SIL-BS	12	ARG114, LEU115, LEU135, TYR138, LEU139, ILE142, ARG145, HIS146, ALA158, TYR161, ARG186, LYS190	−9.315	0.148

**Table 5:** The results of the bio/muco-adhesion tests of the studied compounds.

Sample	Bio-Adhesion		Muco-Adhesion	
	Adhesion Force (N)	Work of Adhesion (mJ)	Adhesion Force (N)	Work of Adhesion (mJ)
SIL M	0.11539 ± 0.00911	0.00857 ± 0.00085049	0.04778 ± 0.00247	0.00623 ± 0.00215
SIL-BS	0.16367 ± 0.02607	0.03117 ± 0.0024	0.09283 ± 0.01178	0.013 ± 0.000655744

**Table 6:** The results of anti-microbial activity of the tested compounds SIL M and SIL BS.

Sample	MIC ^a^ (µg/mL)				
	Fungi			Bacteria	
	*Aspergillus fumigatus*	*Penicillium chrysogenum*	*Fusarium*	*Bacillus* sp.	*Pseudomonas* sp.
SIL M	1.20 ± 0.02	1.20 ± 0.01	1.21 ± 0.01	2.80 ± 0.01	2.90 ± 0.11
SIL-BS 1%	2.08 ± 0.21	1.90 ± 0.12	2.08 ± 0.11	4.04 ± 0.21	4.08 ± 0.24
SIL-BS 25%	>32	>32	>32	>256	>256
Caspofungin ^b^	0.72 ± 0.01	0.72 ± 0.01	0.72 ± 0.01	-	-
Kanamycin ^b^	-	-	-	1.8 ± 1.11	1.6 ± 0.98

^a^ Minimum Inhibitory Concentration (MIC); ^b^ standard compounds.

**Table 7:** Contacting receptor residues by ligand in docked complex M^PRO^@SIL-M, and calculated affinity estimators (relative binding energy and dissociation constant).

Docked Complex (Receptor@Ligand)	No. of Contacting Residues	Contacting Residues in the Receptor (M^PRO^)	*E_b_* (kcal/mol)	*K_d_* (µM)
M^PRO^@SIL M	14	PHE8, LYS102, GLN110, THR111, PHE112, GLN127, ASN151, ILE152, ASP153, SER158, THR292, PHE294, ASP295, ARG298	−5.794	56.61
Remdesivir *			−7.17	
Hidroxychloroquine *			−6.68	
Chloroquine *			−6.47	

* [66].

## Data Availability

Not applicable.

## Supplementary Materials

The following supporting information can be downloaded at: https://www.mdpi.com/article/10.3390/pharmaceutics14122838/s1, Figure S1: IR spectrum of SIL-BS; Figure S2: ^1^H NMR spectrum of SIL-BS; Figure S3: ^13^C NMR spectrum of SIL BS; Figure S4: H, H COSY NMR spectrum of SIL-BS; Figure S5: HSQC NMR spectrum of SIL BS; Figure S6: HMBC NMR spectrum of SIL-BS; Figure S7: UV-vis spectra of the SIL-BS compound in solvents with different polarities showing the keto-enol tautomerism; Figure S8: UV-vis spectra of SIL-BS in PBS (1% DMSO) at pH 1.5, 2.6, 5 si 7.4 during 7 days; Figure S9: ^1^HNMR spectra of the SIL-BS at pH 1–4; Figure S10: Cell viability of SIL M and SIL-BS on NHDF cell lines in comparison with cisplatin; Figure S11: ^1^HNMR spectra of the SIL-BS before, after the addition of protein (BSA) and after 5 days of incubation of the mixture; Figure S12: H, H COSY NMR spectrum of SIL-BS/protein mixture; Table S1: Bond distances (Å) and angles (°) for SIL-BS. The cif and check cif for the compound that is the object of the study were also uploaded.
