# Supplementary material for: Chemical Attachment of 5-Nitrosalicylaldimine Motif to Silatrane Resulting in an Organic–Inorganic Structure with High Medicinal Significance
Source: Pharmaceutics. 2022 Dec 18;14(12):2838. doi: 10.3390/pharmaceutics14122838 (PMC9781643; doi:10.3390/pharmaceutics14122838)
Supplement: Supplementary file 1 [file pharmaceutics-14-02838-s001.zip › pharmaceutics-2066643-supplementary.pdf]

Supplementary Materials

Chemical attachment of 5-nitrosalicylaldimine motif to silatrane resulting in an organic-inorganic structure of high medicinal significance

Mirela-Fernanda Zaltariov, Mihaela Turtoi, Dragos Peptanariu, Ana-Maria Macsim, Lilia Clima, Corneliu Cojocaru, Nicoleta Vornicu, Bianca-Iulia Ciubotaru, Alexandra Bargan, Manuela Calin, Maria Cazacu

Table S1. Bond distances (Å) and angles (°) for **SIL BS.**

Si1-O1	1.657(3)	N2-C9	1.447(9)
Si1-O2	1.665(4)	N3-C13	1.430(11)
Si1-O3	1.667(4)	C8-C9	1.532(8)
Si1-N1	2.151(4)	C8-C7	1.500(7)
Si1-C7	1.877(5)	C15-C14	1.319(11)
O6-C16	1.241(9)	C15-C16	1.425(11)
O4-N3	1.206(10)	C10-C11	1.397(10)
O1-C2	1.408(6)	C11-C12	1.377(10)
O2-C4	1.422(7)	C11-C16	1.498(9)
O3-C6	1.425(7)	C1-C2	1.476(8)
O5-N3	1.204(10)	C4-C3	1.474(9)
N1-C1	1.447(8)	C13-C14	1.404(10)
N1-C3	1.450(8)	C13-C12	1.342(10)
N1-C5	1.451(7)	C6-C5	1.464(9)
N2-C10	1.271(8)		
O1-Si1-O2	117.2(2)	C7-C8-C9	113.2(5)
O1-Si1-O3	119.3(2)	C14-C15-C16	123.4(7)
O1-Si1-N1	83.11(16)	N2-C10-C11	127.9(6)
O1-Si1-C7	98.3(2)	C10-C11-C16	120.3(7)
O2-Si1-O3	119.0(2)	C12-C11-C10	122.7(7)
O2-Si1-N1	82.99(17)	C12-C11-C16	117.0(8)
O2-Si1-C7	97.8(2)	N1-C1-C2	108.9(5)
O3-Si1-N1	82.69(18)	O1-C2-C1	110.8(5)
O3-Si1-C7	95.2(2)	O2-C4-C3	110.2(5)
C7-Si1-N1	177.8(2)	C14-C13-N3	121.6(9)
C2-O1-Si1	123.4(3)	C12-C13-N3	118.5(8)
C4-O2-Si1	123.1(3)	C12-C13-C14	119.9(8)
C6-O3-Si1	123.2(4)	O3-C6-C5	109.8(5)
C1-N1-Si1	105.1(3)	N1-C3-C4	108.8(5)
C1-N1-C3	112.4(6)	N1-C5-C6	109.0(5)
C1-N1-C5	114.2(6)	C15-C14-C13	120.3(9)

C3-N1-Si1	105.3(3)	N2-C9-C8	112.6(5)
C3-N1-C5	113.6(6)	C8-C7-Si1	117.0(4)
C5-N1-Si1	105.1(4)	C13-C12-C11	123.8(7)
C10-N2-C9	124.9(5)	O6-C16-C15	124.3(7)
O4-N3-C13	121.6(9)	O6-C16-C11	120.2(8)
O5-N3-O4	121.1(10)	C15-C16-C11	115.5(7)
O5-N3-C13	117.2(10)		

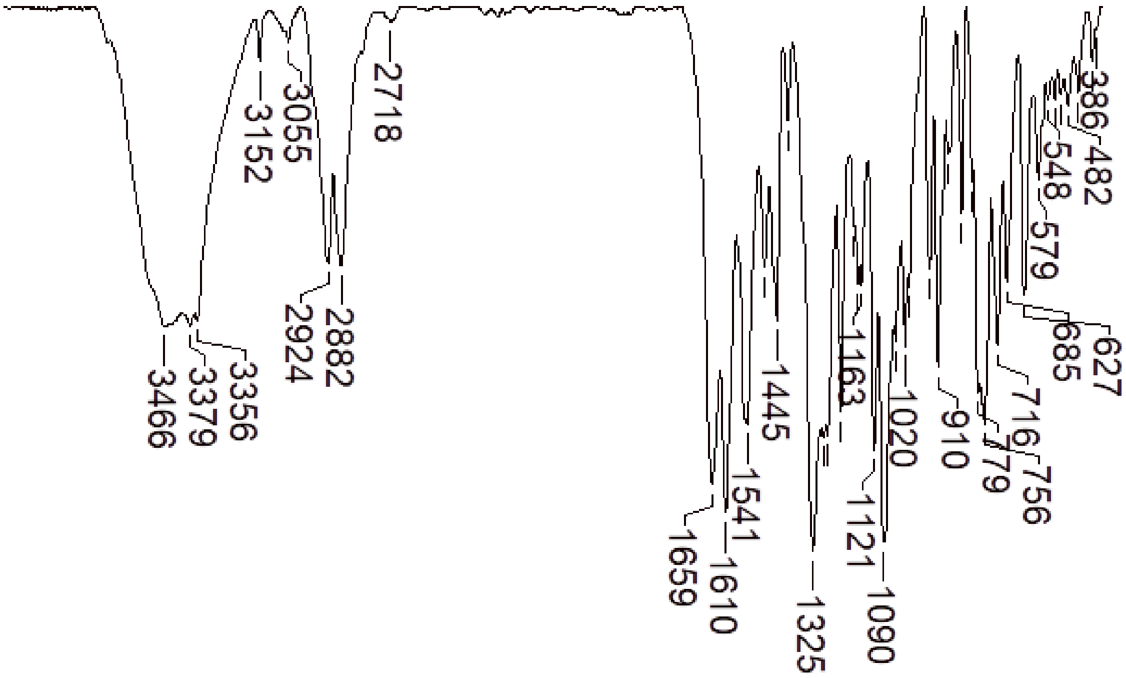

Figure S1. IR spectrum of SIL-BS.

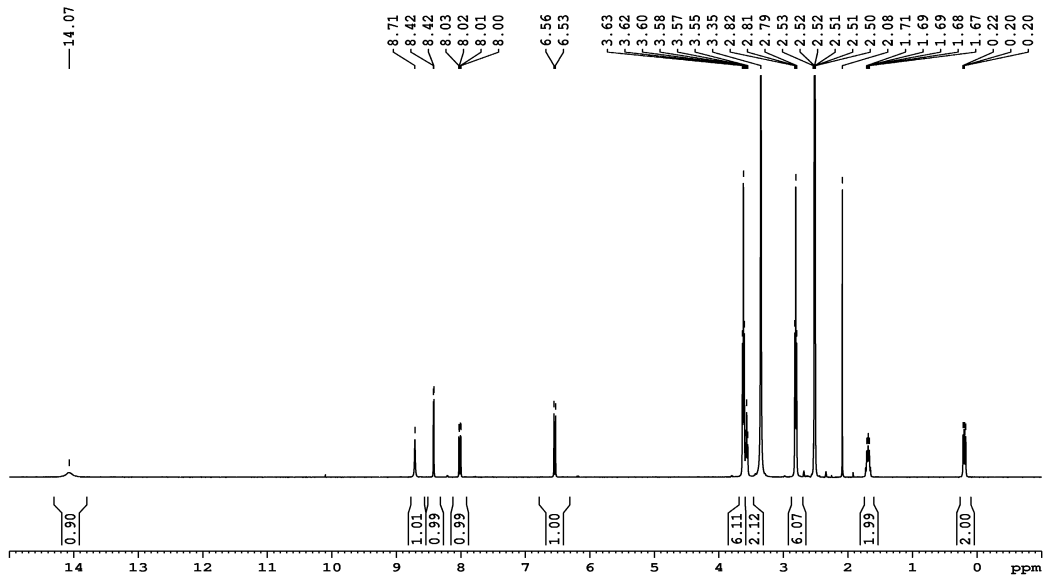

Figure S2. ^{1}H NMR spectrum of SIL-BS.

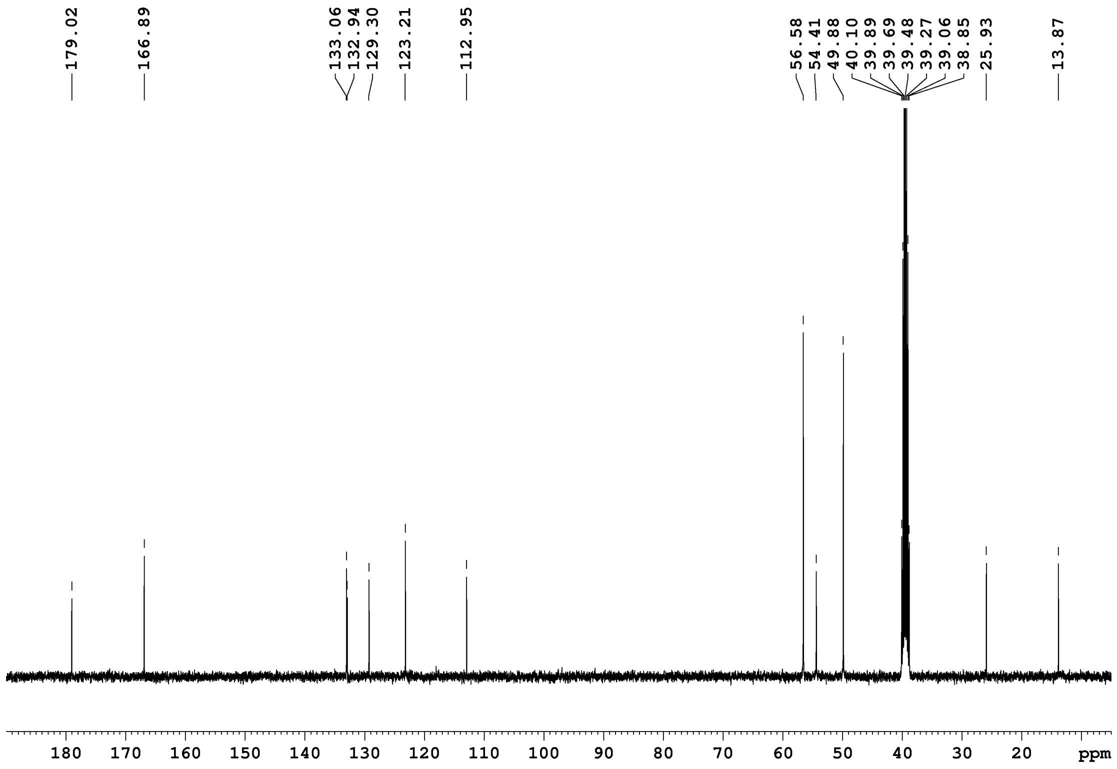

Figure S3. ^{13}C NMR spectrum of SIL-BS.

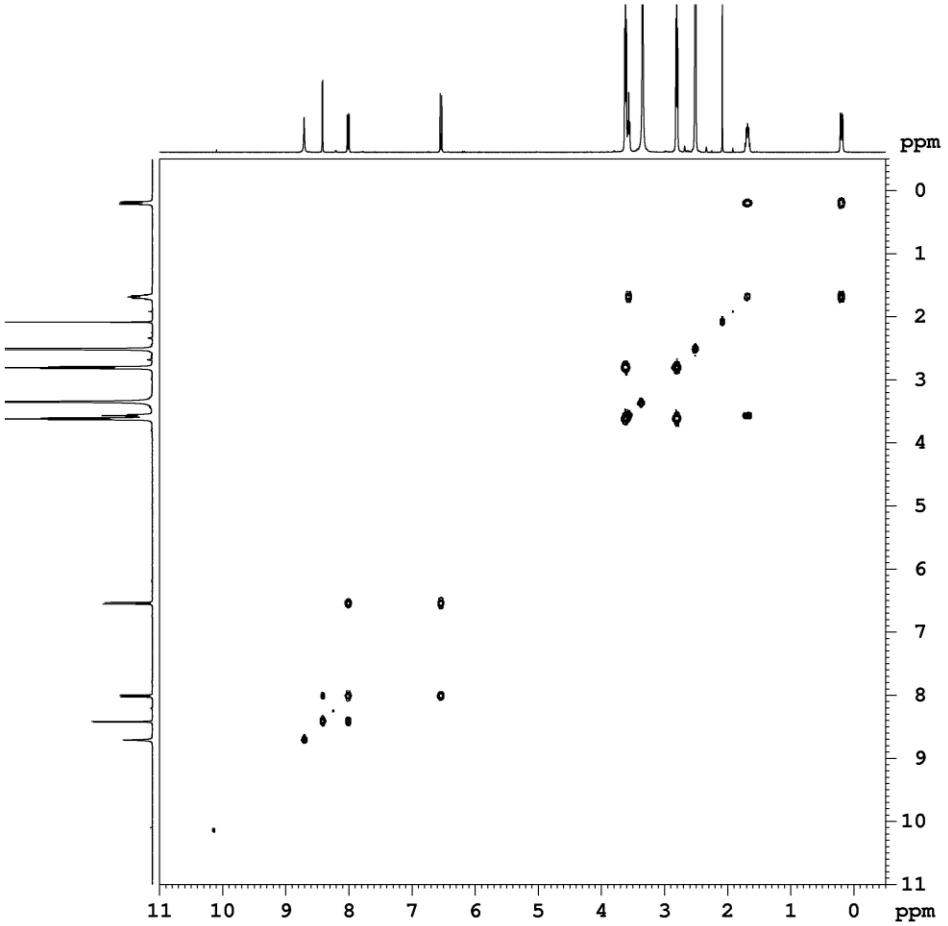

Figure S4. H, H COSY NMR spectrum of SIL-BS.

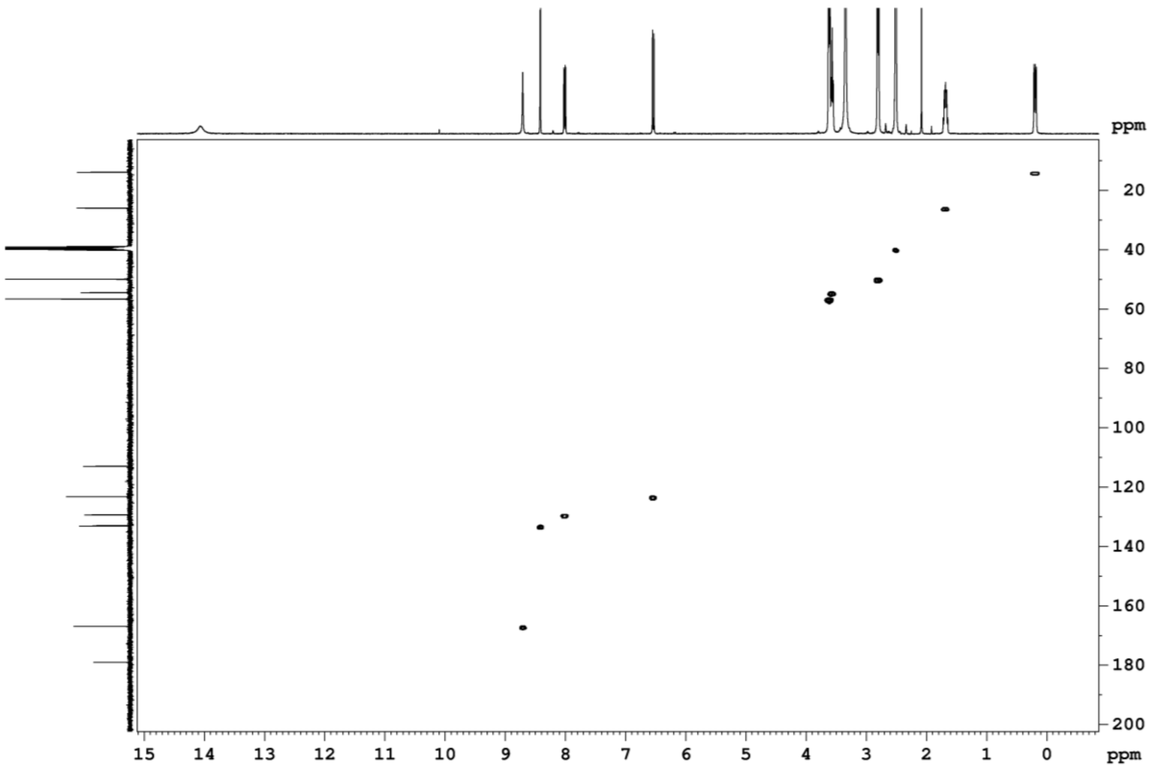

Figure S5. HSQC NMR spectrum of SIL-BS.

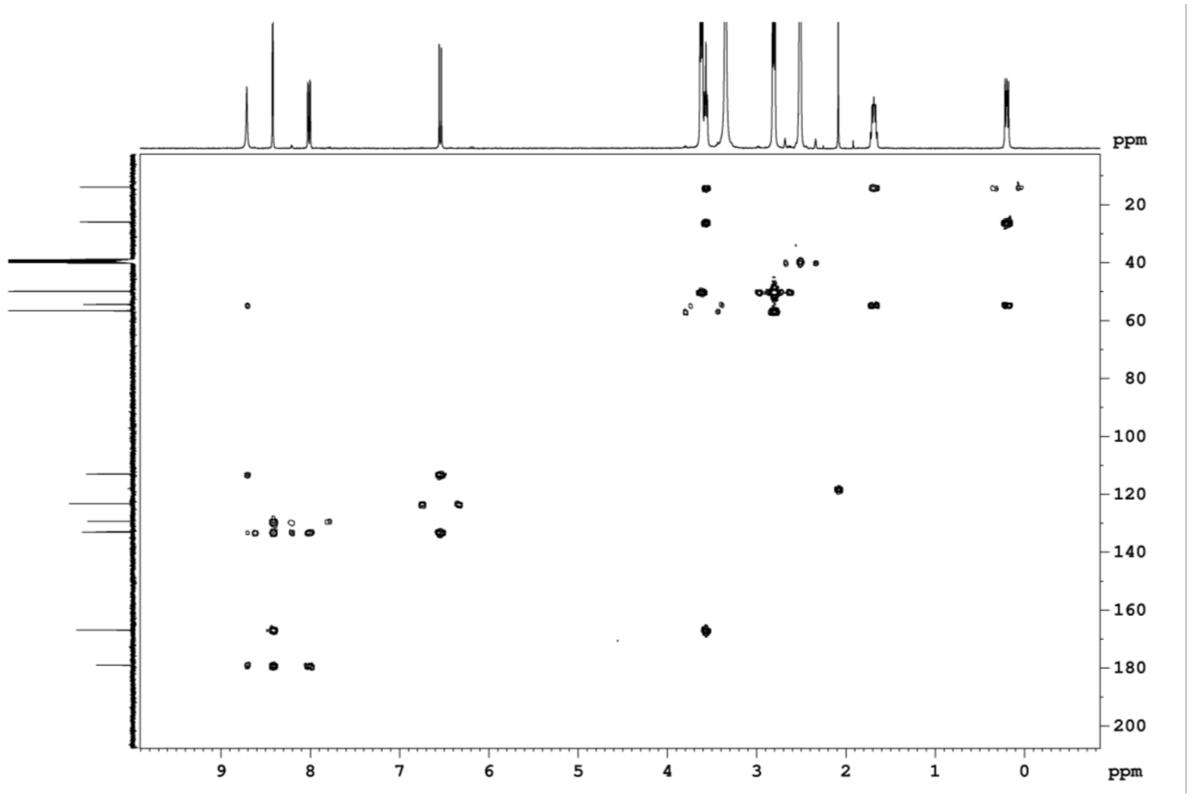

Figure S6. HMBC NMR spectrum of SIL-BS.

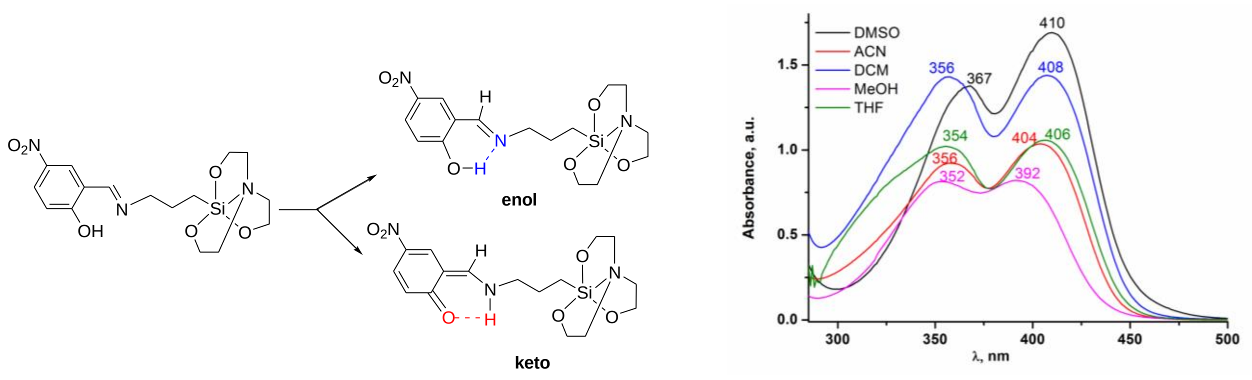

Figure S7. UV-vis spectra of the SIL-BS compound in solvents with different polarities showing the keto-enol tautomerism.

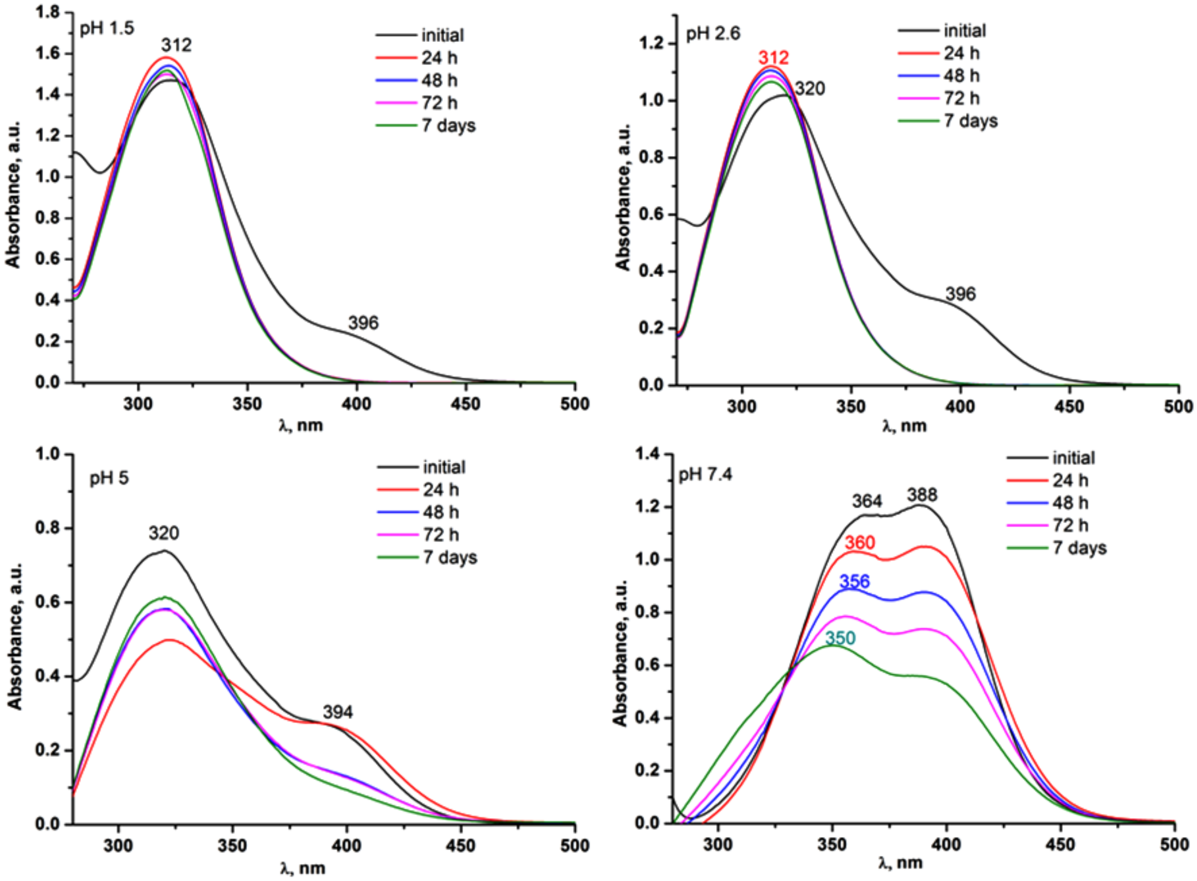

Figure S8. UV-vis spectra of SIL-BS in PBS (1% DMSO) at pH 1.5, 2.6, 5 si 7.4 during 7 days.

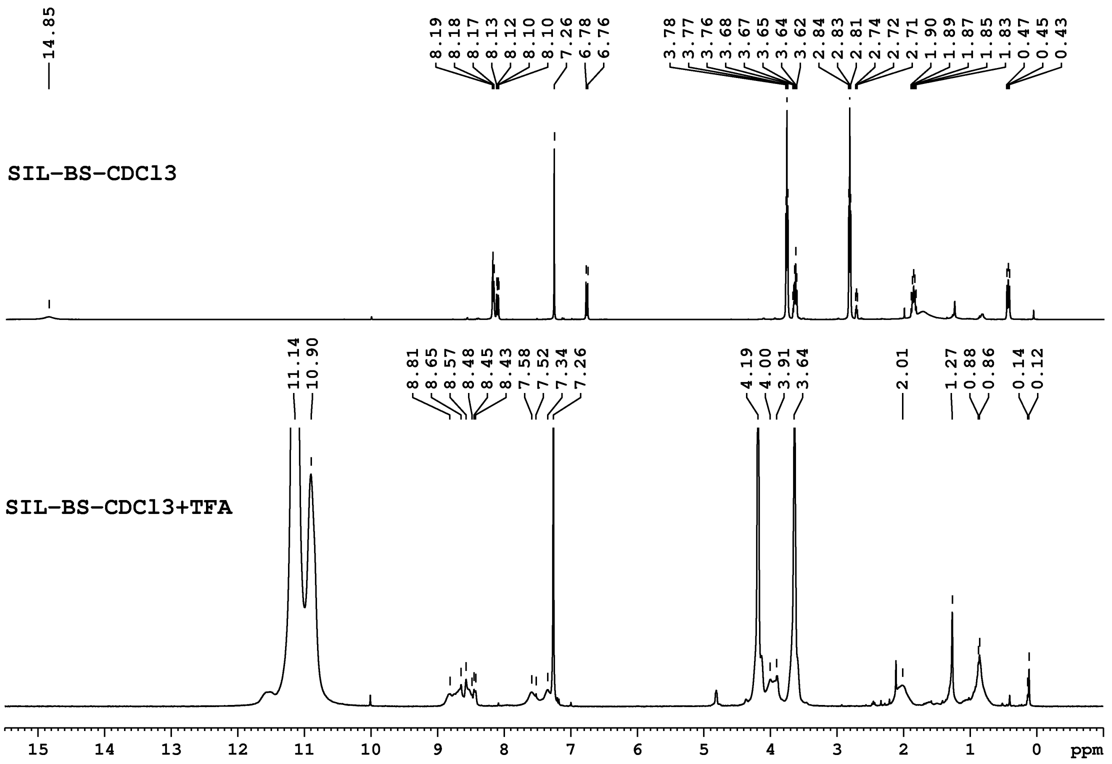

Figure S9. ^{1}HNMR spectra of the SIL-BS at pH 1-4.

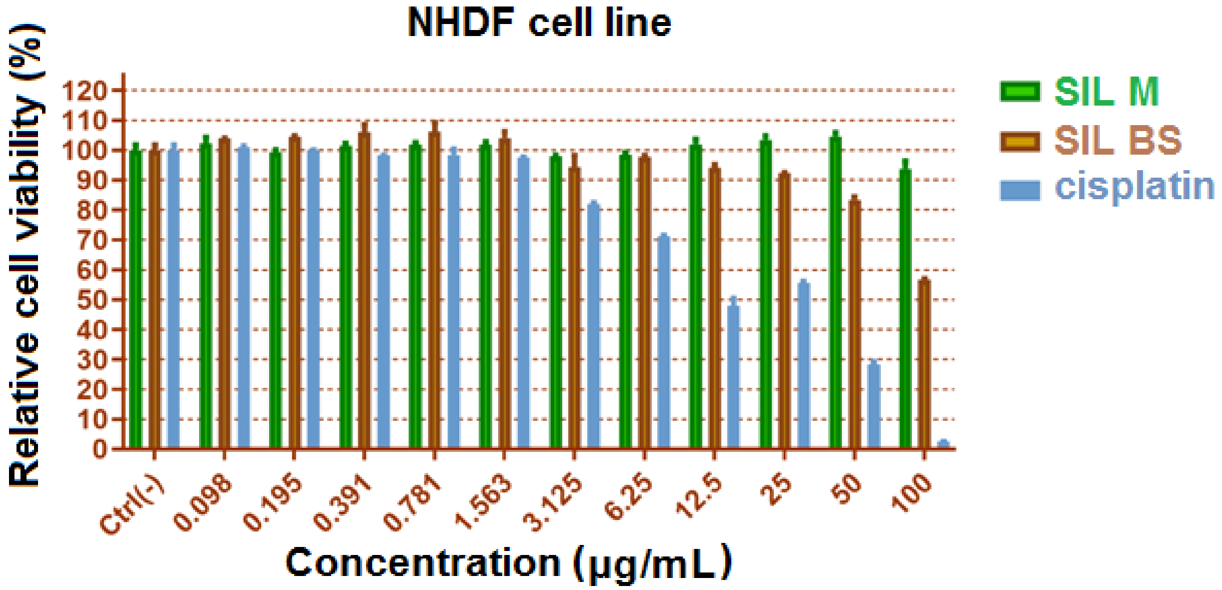

Figure S10. Cell viability of SIL M and SIL-BS on NHDF cell lines in comparison with cisplatin.

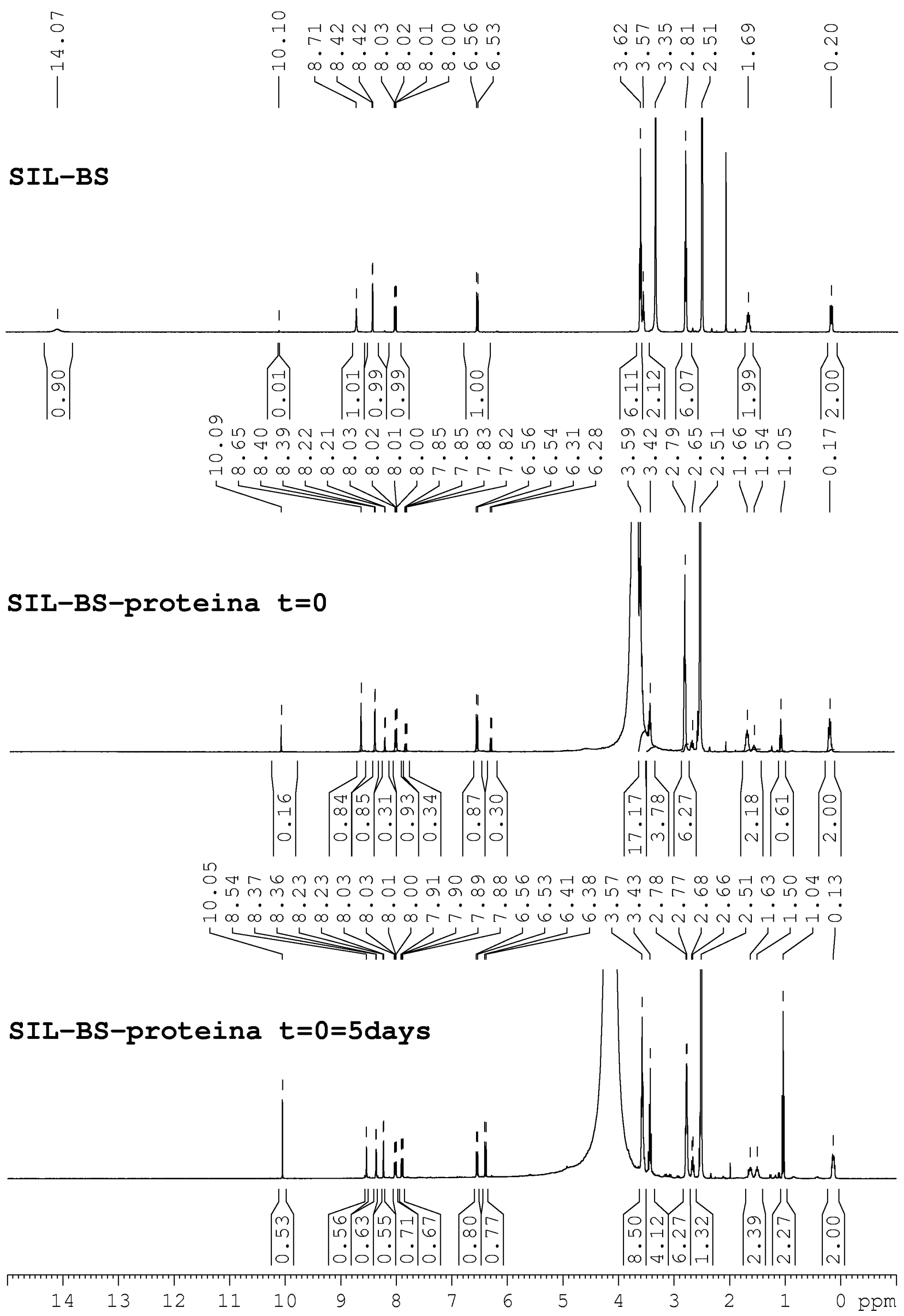

Figure S11. ^{1}H NMR spectra of the SIL BS before, after the addition of protein (BSA) and after 5 days of incubation of the mixture.

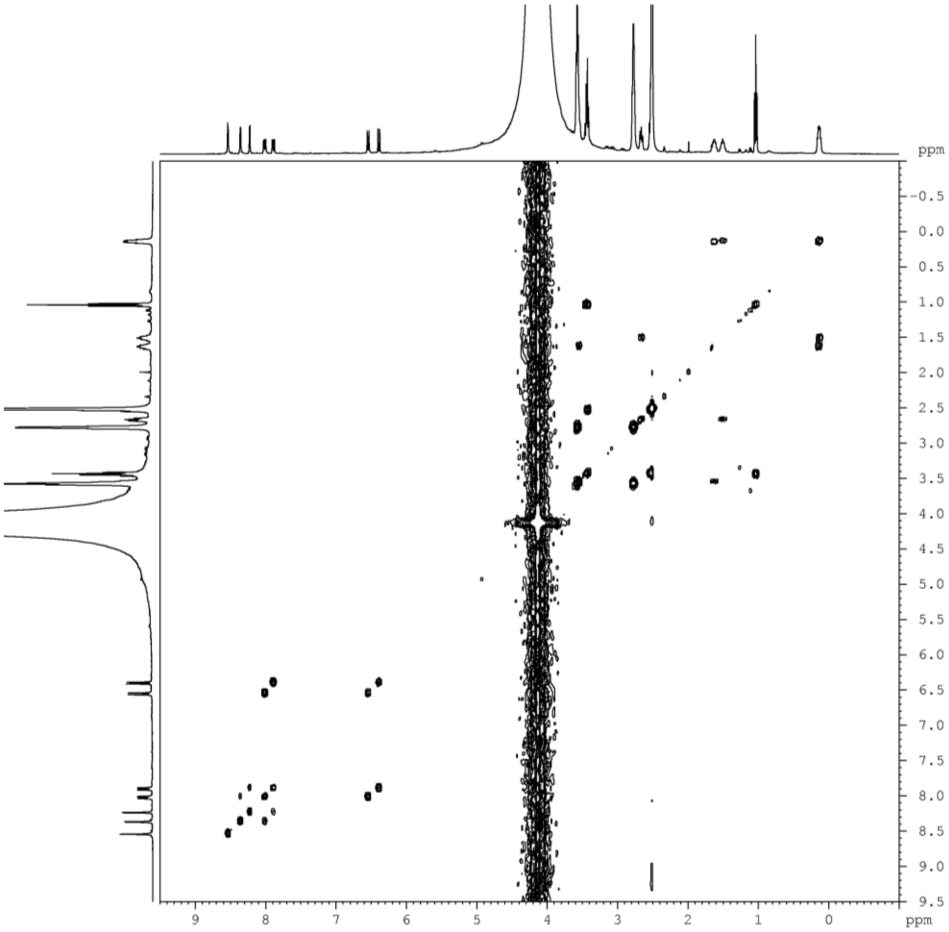

Figure S12. H, H COSY NMR spectrum of SIL-BS/protein mixture.
